# A Holistic Investigation of *Arabidopsis* Proteomes Altered in Chloroplast Biogenesis and Retrograde Signalling Identifies PsbO as a Key Regulator of Chloroplast Quality Control

**DOI:** 10.1111/pce.15611

**Published:** 2025-05-14

**Authors:** Dario Di Silvestre, Nicolaj Jeran, Guido Domingo, Candida Vannini, Milena Marsoni, Stefania Fortunato, Maria Concetta de Pinto, Alberto Tamborrino, Yuri Luca Negroni, Michela Zottini, Lien Tran Hong, Andrea Lomagno, Pierluigi Mauri, Paolo Pesaresi, Luca Tadini

**Affiliations:** ^1^ Institute of Biomedical Technologies National Research Council Segrate Italy; ^2^ Dipartimento di Bioscienze Università degli Studi di Milano Milano Italy; ^3^ Dipartimento di Biotecnologie e Scienze della Vita Università degli Studi dell'Insubria Varese Italy; ^4^ Department of Biosciences, Biotechnology and Environment University of Bari Aldo Moro Bari Italy; ^5^ Department of Biology University of Padova Padova Italy

**Keywords:** chloroplast biology, chloroplast degradation, intracellular signalling, oxidative stress, proteome, proteomics

## Abstract

Communication between the diverse compartments of plant cells relies on an intricate network of molecular interactions that orchestrate organellar development and adaptation to environmental conditions. Plastid‐to‐nucleus signalling pathways play a key role in relaying information from developing, mature, and damaged or disintegrating chloroplasts to the nucleus, which serves to coordinate gene expression between the two genomes. To shed light on these mechanisms, we performed a comprehensive analysis of the response of the *Arabidopsis thaliana* proteomes to perturbation of chloroplast biogenesis by the antibiotic lincomycin (Lin) in the absence of GENOMES UNCOUPLED 1 (GUN1), a key player in plastid‐to‐nucleus signalling. The topological analysis of protein–protein interactions (PPIs) and co‐expression networks enabled the identification of protein hubs in each genotype and condition tested, and highlighted whole‐cell adaptive responses to the disruption of chloroplast biogenesis. Our findings reveal a novel role for PsbO, a subunit of the oxygen‐evolving complex (OEC), which behaves as an atypical photosynthetic protein upon inhibition of plastid protein synthesis. Notably, and unlike all other subunits of the thylakoid electron transport chain, PsbO accumulates in non‐photosynthetic plastids, and is crucial for the breakdown of damaged chloroplasts.

## Summary statement

- The holistic analysis of *Arabidopsis* proteomes revealed PsbO, a subunit of the oxygen‐evolving complex, as a key regulator of chloroplast quality control.
- PsbO accumulates in non‐photosynthetic plastids and exerts a novel role in plastid homoeostasis.

## 1 Introduction

Unravelling the mechanisms that govern communication between plant‐cell organelles and compartments is a key step in elucidating the basis for plant adaptation to environmental changes, and for developing strategies that increase plant yield and stress tolerance (Wu and Bock 2021). Chloroplasts and mitochondria are important players in sensing developmental and environmental stimuli, and conveying this information to the nucleus (Y. Wang et al. 2020). Signals passed between nucleus and organelles (i.e., anterograde and retrograde communication) are integrated into a complex network of interactions, and play a crucial role in regulating developmental stages, adaptation processes, and the functional status of organelles (Ng et al. 2014; Wu and Bock 2021). During the last two decades, interest in anterograde and retrograde communication has increased, and substantial progress has been made in identifying key components and pathways involved. For instance, upon accumulation of unfolded or misfolded proteins in either organelle, mitochondrial (mtUPR) (X. Wang and Auwerx 2017) or chloroplast (cpUPR) (Llamas et al. 2017) Unfolded Protein Response(s) are activated, which result in increased transcription of protective genes, including those that encode chaperones and proteases (Richter et al. 2023). Many putative signalling molecules, such as carotenoids (Woodson et al. 2011; Moreno et al. 2021), metabolites (Milanesi et al. 2020; Fang et al. 2019; Vogel et al. 2014), intermediates of tetrapyrrole biosynthesis (Woodson et al. 2011), isoprenoid precursors (Xiao et al. 2012), nucleotides (S. Ono, Suzuki, et al. 2021; Estavillo et al. 2011) and redox signals (Jan et al. 2022; Maruta et al. 2012), have been identified. Moreover, the protein GENOMES UNCOUPLED 1 (GUN1) has been shown to be a key mediator of chloroplast retrograde communication, which affects proteins and processes such as the cytosolic HSP90 complex, protein import capacity and folding stress (Wu, Meyer, Richter et al. 2019; Tadini, Peracchio, et al. 2020). Other studies have depicted GUN1 as a fail‐safe factor during the critical step of seedling emergence from darkness, during which it regulates (i) transcription factors involved in light responses, photomorphogenesis and chloroplast development (Hernández‐Verdeja et al. 2022; Veciana et al. 2022; Wu, Meyer, Richter et al. 2019); (ii) the redox changes that occur during biogenic retrograde signalling (Fortunato et al. 2022), and (iii) the acquisition of basal thermotolerance in *Arabidopsis thaliana* (*A. thaliana*) (Lasorella et al. 2022). Recently, GUN1 has been shown to have conventional PPR protein features, displaying RNA binding capability (Tang et al. 2024), while several studies have depicted it as a stress‐response factor that is regulated at the post‐translational level and is critical for adaptation to environmental challenges (Wu et al. 2018; Tadini, Peracchio, et al. 2020; X. Zhao et al. 2018).

Although the mechanisms of direct communication between chloroplasts and mitochondria are still poorly understood, the existence of common mitochondrion‐ and plastid‐to‐nucleus retrograde signalling pathways has been widely documented (Y. Wang et al. 2020). The identification of numerous chloroplast and mitochondrial dual‐located proteins involved in diverse functions (Xu et al. 2013; Mitschke et al. 2009), together with the notion that chloroplasts and mitochondria have a synergistic effect in regulating nuclear gene expression (Pesaresi et al. 2006), strongly indicate the existence of a tightly integrated network of mitochondrial and chloroplast anterograde and retrograde signalling pathways (Y. Wang et al. 2020). Organelle‐to‐nucleus retrograde communication also requires cytosolic and nuclear components, such as kinases and transcription factors, which are shared between chloroplasts and mitochondria (Wurzinger et al. 2018; Blanco et al. 2014; Shapiguzov et al. 2019; De Clercq et al. 2013; Van Aken and Pogson 2017; Van Aken et al. 2013), and whose accumulation is thought to be influenced by cytosolic folding stress (Wu, Meyer, Richter et al. 2019; Tadini, Jeran, Peracchio et al. 2020). These findings highlight the active roles of cell compartments in organellar quality control and degradation. Importantly, the disassembly of organelles triggers autophagic pathways which, on the one hand, are essential for plant development and appropriate responses to biotic and abiotic stresses and, on the other ensure the maintenance of a functional population of organelles, while promoting nutrient redistribution to sink tissues (Woodson 2022; Van Aken and Van Breusegem 2015).

The intricate signalling networks that govern diverse cellular communication pathways underscore the importance of a comprehensive understanding of the key mechanisms and components involved in organelle‐to‐nucleus and organelle‐to‐organelle interactions. Among the studies that have focused on organelle signalling, some have combined transcriptomic and proteomic profiles (Wu, Meyer, Wu, et al. 2019; Marino et al. 2019), others have taken metabolomics into consideration (Bjornson et al. 2017), while more recent studies were based on proteomics data (Wu et al. 2018, Wu, Meyer, Richter et al. 2019; Tadini, Peracchio, et al. 2020). To take advantage of the plethora of information contained in ‘‐omics’ profiles, we adopted a systems biology approach based on graph theory and network analysis (Di Silvestre et al. 2018), which represents a novelty in the study of plant retrograde signalling and chloroplast homoeostasis. Here, protein profiling and quantification were modelled as protein–protein interactions (PPIs) and co‐expression networks (Jeong et al. 2001; Vella et al. 2017), which were processed to define protein hubs (Di Silvestre et al. 2021, 2022). Following this strategy, we investigated proteomes obtained from seedlings of wild‐type *A. thaliana* (Col‐0), as well as *gun1‐101* and *gun1‐102* mutant plants, which had been treated (or not) with lincomycin (Lin), a chloroplast‐specific translation inhibitor that has been widely used to perturb chloroplast biogenesis and trigger plastid‐to‐nucleus retrograde communication (Koussevitzky et al. 2007; Chotewutmontri and Barkan 2018). Hypotheses and key players that emerged from this holistic approach were then validated experimentally. Our findings show that PsbO, a subunit of the oxygen‐evolving complex (OEC), assumes an unexpected function as a key regulator of the degradation of non‐functional chloroplasts.

## 2 Results

### 2.1 Proteome Profiles of Col‐0 and *gun1* Seedlings Grown in the Presence of Lincomycin

**Figure 1:**
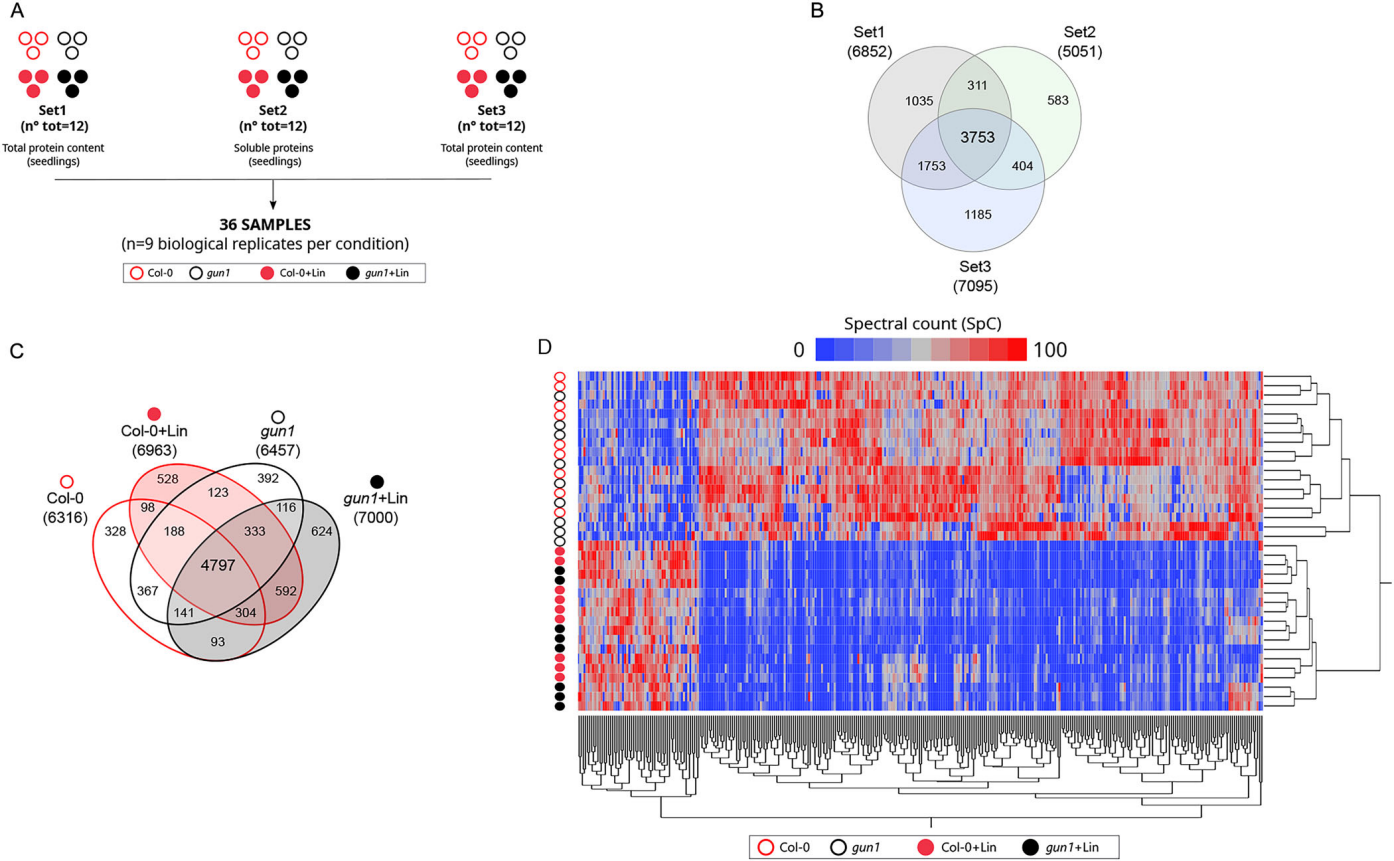
Proteomes of *A. thaliana* Col‐0 and *gun1* seedlings grown on MS medium in the absence or presence (±) of Lin. (A) Schematic overview of the experimental set‐up adopted in this study, which depicts the relevant conditions, data sets and replicates used. Each circle represents one replicate. Empty circles indicate control conditions and filled circles refer to Lin‐grown samples. Col‐0 samples are shown in red, *gun1* in black. (B) Venn diagram of proteins identified in Sets 1, 2 and 3. The data in Set1 were generated in our laboratory from total protein extracts obtained from Col‐0 ± Lin and *gun1‐102* ± Lin seedlings harvested 6 days after sowing (DAS). Set2 was derived from Col‐0 ± Lin and *gun1‐102* ± Lin seedlings, 6 DAS, and is enriched in soluble proteins (Tadini, Peracchio, et al. 2020), while Set3 was obtained from total protein extracts from Col‐0 ± Lin; *gun1‐101* ± Lin seedlings, 5 DAS (Wu, Meyer, Wu, et al. 2019). (C) Venn diagram showing the proteins identified in the three data sets [refer to the shape/colour code shown in (A)] and grouped according to the four conditions: Col‐0 and the *gun1* mutant grown in the presence or absence of Lin (±Lin). (D) Hierarchical clustering of differentially abundant proteins (DAPs), *p* ≤ 0.001.

To investigate adaptive responses triggered by the inhibition of chloroplast biogenesis in relation to plastid‐to‐nucleus communication, we integrated and analyzed three independent proteome data sets (Set1, Set2, and Set3) derived from *A. thaliana* Col‐0 and *gun1* seedlings grown in the absence or presence of Lin (±Lin) (Figure 1A). Set2 (Tadini, Peracchio, et al. 2020) and Set3 (Wu, Meyer, Wu, et al. 2019) were previously described and have been reprocessed alongside Set1, which was specifically generated to expand and further strengthen the proteomics data and their analyses. Large numbers of proteins were identified in Set1 (6852; this study) and Set3 (7095; Wu, Meyer, Wu, et al. 2019), both of which contained proteins from total seedling extracts. A slightly lower number was identified in Set2, which was enriched in soluble proteins (5051; Tadini, Peracchio, et al. 2020). Overall, 3753 proteins were in common among the three data sets, regardless of the conditions. (Figure 1B). Altogether, 9024 distinct proteins were identified by combining 36 LC–MS/MS runs, while a comparable number was found under each condition and a total of 4797 proteins were shared across the four conditions (Figure 1C). Among the 9024 proteins identified in all LC–MS/MS runs, 837 were common to all of the 36 samples analyzed (Supporting Information S11: Table S1). The correlations among the global protein profiles revealed a higher degree of divergence between proteomes of seedlings grown in the absence/presence of Lin than between Col‐0 and *gun1* genotypes, and this was also true for the two +Lin growth conditions (Supporting Information S1: Figure S1). A similar pattern was observed following a preliminary analysis of Gene Ontology (GO) term enrichment. Indeed, most Biological Processes (BPs) were significantly reduced in seedlings exposed to Lin relative to control conditions, regardless of their genetic background. Among the BP terms assessed, those associated with chloroplast homoeostasis, functionality and energy production were either strongly reduced or absent (Supporting Information S2: Figure S2 and Supporting Information 12: Table S2). In contrast to this result, BP terms associated with Development/Morphogenesis, RNA processing, and Nuclear and Golgi transport were increased, possibly indicating active cellular and nuclear reprogramming (Supporting Information S2: Figure S2). BPs linked to mitochondrial organization, and ATP synthesis coupled with electron transport, were also more prominent, suggesting a potential compensatory role for mitochondria in response to chloroplast impairment (Supporting Information S2: Figure S2). The Molecular Function (MF) terms mirrored the enrichment analysis of BPs, highlighting a reduction in tetrapyrrole binding and translation‐related RNA binding. Notably, the Folding/Response to Stress terms were more prominently associated with the *gun1* + Lin proteome (Supporting Information S2: Figure S2).

**Figure 2:**
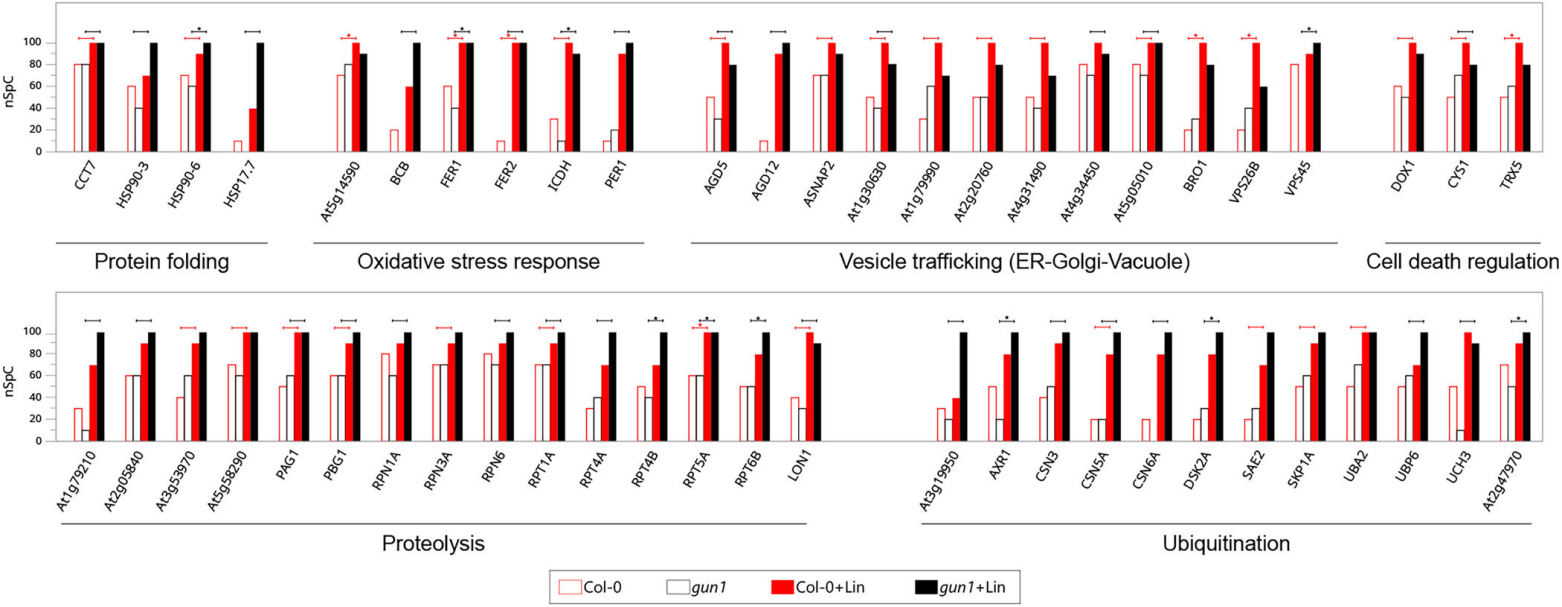
Selection of DAPs upregulated in the presence of Lin. These proteins are functionally involved in protein folding, oxidative stress response, vesicle trafficking (ER–Golgi–Vacuole), cell death regulation, proteolysis and ubiquitination. For each protein, the normalized spectral count value (nSpC) in the range 0–100 is shown. Highlighted comparisons have a statistical significance of *p* ≤ 0.05, while asterisks indicate a significance of *p* ≤ 0.01, calculated according to LDA. [Color figure can be viewed at wileyonlinelibrary.com]

Label‐free quantitative comparison of the characterized proteomes identified 745 differentially abundant proteins (DAPs; *p* ≤ 0.01; see Figure 1D and Supporting Information S13: Table S3). The largest differences were observed upon comparison of Col‐0 versus Col‐0 + Lin (537 DAPs) and of *gun1* versus *gun1* + Lin (547 DAPs; Figure 1D and Supporting Information S1: Figure S1), implying a stronger effect of lincomycin‐mediated inhibition of plastid translation than that caused by the *gun1* mutation. To obtain a comprehensive representation, high‐confidence DAPs (*p* ≤ 0.001, *n* = 326) were modelled as PPI networks and grouped into 41 functional modules representing pathways, GO BPs, GO MFs, GO Cellular Components (CCs) and protein families (Supporting Information S3: Figure S3). As expected, the levels of accumulation of most functional modules collapsed in both genotypes upon the inhibition of plastid translation (+Lin). Conversely, functional modules enriched in protein folding, ubiquitination, proteolysis, the TCA cycle, and lipid and carbohydrate metabolism, respectively, were upregulated upon treatment with Lin (Supporting Information S3: Figure S3). Since the reduced accumulation of large protein complexes and metabolic pathways are interpretable as the consequence of Lin‐mediated inhibition of chloroplast biogenesis, we focused on proteins whose abundance was either unchanged or increased upon Lin treatment. When grouped by function, these proteins revealed marked increases in stress‐related adaptive responses and cellular remodelling. The polypeptides involved included peptidases, proteases, and processes such as ubiquitination, misfolded protein degradation, protein folding, cell death regulation, vesicle transport, organelle transport, vacuolar processes, fatty acid metabolism and oxidative stress response (Figure 2 and Supporting Information S3: Figure S3). Specific protein families were enriched, such as serine carboxypeptidase (SCPL) in the peptidase module (Havé et al. 2018; J. Huang et al. 2008) and the regulatory particle ATPase (RPT), as well as non‐ATPase (RPN) subunits of the 26S proteasome in the protease module. In addition, some proteins were exclusively detected in the proteomes of Col‐0 and *gun1* seedlings grown in the presence of Lin. These included (i) acetyl‐CoA carboxylase 2 (ACC2), which is involved in fatty‐acid metabolism and is regulated by GUN1‐dependent retrograde signalling (Parker et al. 2014, 2016; J. Wang et al. 2018; Perez de Souza et al. 2020); (ii) the peptidase serine carboxypeptidase‐like 9 (SCPL9), and the nuclear‐pore‐complex protein NUP62, which is associated with nuclear protein transport (Kemp et al. 2015; Q. Zhao and Meier 2011; Supporting Information S4: Figure S4).

### 2.2 Hubs From PPI and Co‐Expression Network Models

**Figure 3:**
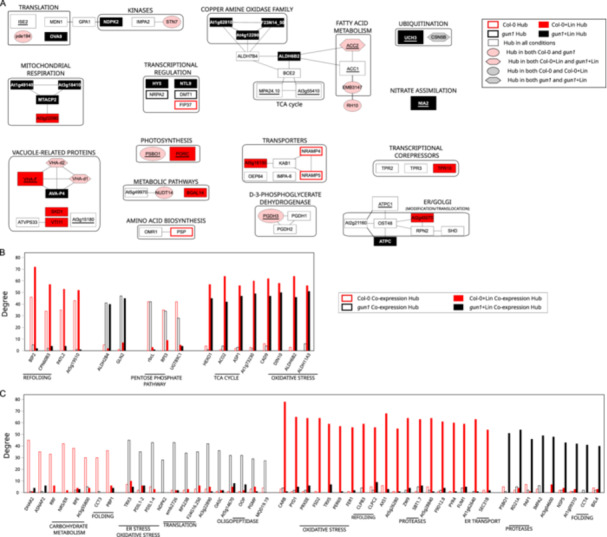
Hubs derived from PPI and co‐expression network models. (A) PPI network hubs in Col‐0 and *gun1* seedlings, grown in the absence or presence of Lin (±Lin). PPI hubs were selected on the basis of betweenness, centroid and bridging centralities. Larger and underlined nodes indicate proteins that were defined as hubs and were differentially abundant (*p* ≤ 0.01). (B) Hub proteins based on co‐expression network models and genetic background or growth condition. (C) Hub proteins exclusively found in Col‐0, *gun1*, Col‐0 + Lin or *gun1* + Lin co‐expression network models. [Color figure can be viewed at wileyonlinelibrary.com]

The characterized proteome profiles were modelled as PPIs and protein co‐expression networks, and were processed at the topological level and hubs were identified for each genotype and growth condition. All four models showed scale‐free distribution. Protein hubs were selected by combining betweenness, centroid and bridging centralities, and their significance was assessed using random networks that showed significantly different average betweenness values (Supporting Information S5: Figure S5). Globally, 66 proteins were defined as hubs, 25 of them in all conditions, while 41 were genotype/growth condition‐specific (Figure 3A and Supporting Information S14: Table S4). Photosynthesis‐ and vacuole‐related proteins, including the vacuolar ATPase complex, were prominent hubs predominantly found in Col‐0 + Lin seedlings. The vacuole‐localized hubs SKD1 and VTI11, which mediate the trafficking and delivery of cargos to lytic vacuoles (Muntz 2007; Sanmartín et al. 2007; Shahriari et al. 2010), highlight the vacuole's significant role in cytosolic clearance in response to Lin treatment. In *gun1*, and especially in *gun1* + Lin seedlings, identified protein hubs are involved in the mitochondrial respiratory chain, ubiquitination, transcriptional regulation, or belong to the copper amine oxidase family. The double relevance of proteins that were found to be hubs and to be differentially accumulated also emerged. Among others, Acetyl‐CoA Carboxylase 1 (ACC1), which participates in fatty‐acid biosynthesis (Damiano et al. 2018), was found to serve as a hub in both genetic backgrounds under Lin‐growth conditions and was upregulated in the same conditions. The subunit of the OEC PsbO1 (Lundin et al. 2007; Jiang et al. 2017) behaved as a hub in untreated seedlings and was downregulated upon growth in Lin‐containing medium (Figure 3A and Supporting Information S11: Table S1). Moreover, nitrate reductase [NADH] 2 (NIA2), which acts in nitrate assimilation and detoxification of reactive oxygen species (ROS) and reactive nitrogen species (RNS) (Manbir et al. 2022; Khator and Shekhawat 2020; Pan et al. 2019), was a hub in *gun1* + Lin seedlings. Furthermore, NIA2 accumulation was not altered in *gun1* + Lin relative to the untreated controls, while it was found reduced in Col‐0 + Lin compared to Col‐0 samples (Supporting Information S11: Table S1).

Implementation of the hub description was achieved by integrating the topological analysis of the co‐expression networks (Supporting Information S15: Table S5). Similarly to PPI hubs, their significance was assessed in terms of the degree of centrality, calculated using random networks (Supporting Information S5: Figure S5). In both genetic backgrounds, upon growth on Lin, a high degree of correlation was observed between proteins involved in oxidative stress and the TCA cycle (Figure 3B,C), in good agreement with the enriched GO terms (Supporting Information S2: Figure S2). Interestingly, proteins involved in protein refolding, such as the ER‐located heat shock 70 kDa protein (BIP2; Srivastava et al. 2013; Yang et al. 2014) and the plastid‐located Chaperonin 60 subunit beta 3 (CPN60B3; Klasek et al. 2020), were strongly correlated in Col‐0, and to an even higher degree in Col‐0 + Lin. Conversely, the mitochondrial aldehyde dehydrogenase ALDH2B4 (Skibbe et al. 2002) and the dual‐located chloroplast/mitochondria glutamine synthetase 2 (GLN2; Taira et al. 2004) were hubs in *gun1* samples under both conditions (Figure 3B). Interestingly, among the coregulated hubs found upon Lin treatment that were uniquely correlated in either Col‐0 or *gun1* genotypes, 11 out of the 19 hubs found in Col‐0 + Lin samples were located in the plastid (Figure 3C and Supporting Information S15: Table S5). Notably, this subgroup included stress‐response proteins—namely, the unfoldase ClpB3 (Llamas et al. 2017; Parcerisa et al. 2020), the chaperone ClpC2 (Park and Rodermel 2004) and two ROS‐scavenger enzymes (PRXIIE and FSD2). On the other hand, among the highly correlated hubs detected in *gun1* + Lin, the proteasome subunit PAF1 (Sung et al. 2009) and the RESPONSIVE TO DEHYDRATION 21 A (RD21A) protein, which are involved in cytosolic protein degradation and vacuolar‐stress‐mediated cell death (Koh et al. 2016; Boex‐Fontvieille et al. 2015; Z. Wang et al. 2008), respectively, were identified. Strikingly, all *gun1* + Lin coregulation hubs were extra‐plastid proteins, with the sole exception of Oxygen‐evolving enhancer protein 1‐1 (PsbO1), which is involved in water oxidation in Photosystem II.

### 2.3 Functional Validation of Hubs From PPI and Co‐Expression Networks

**Figure 4:**
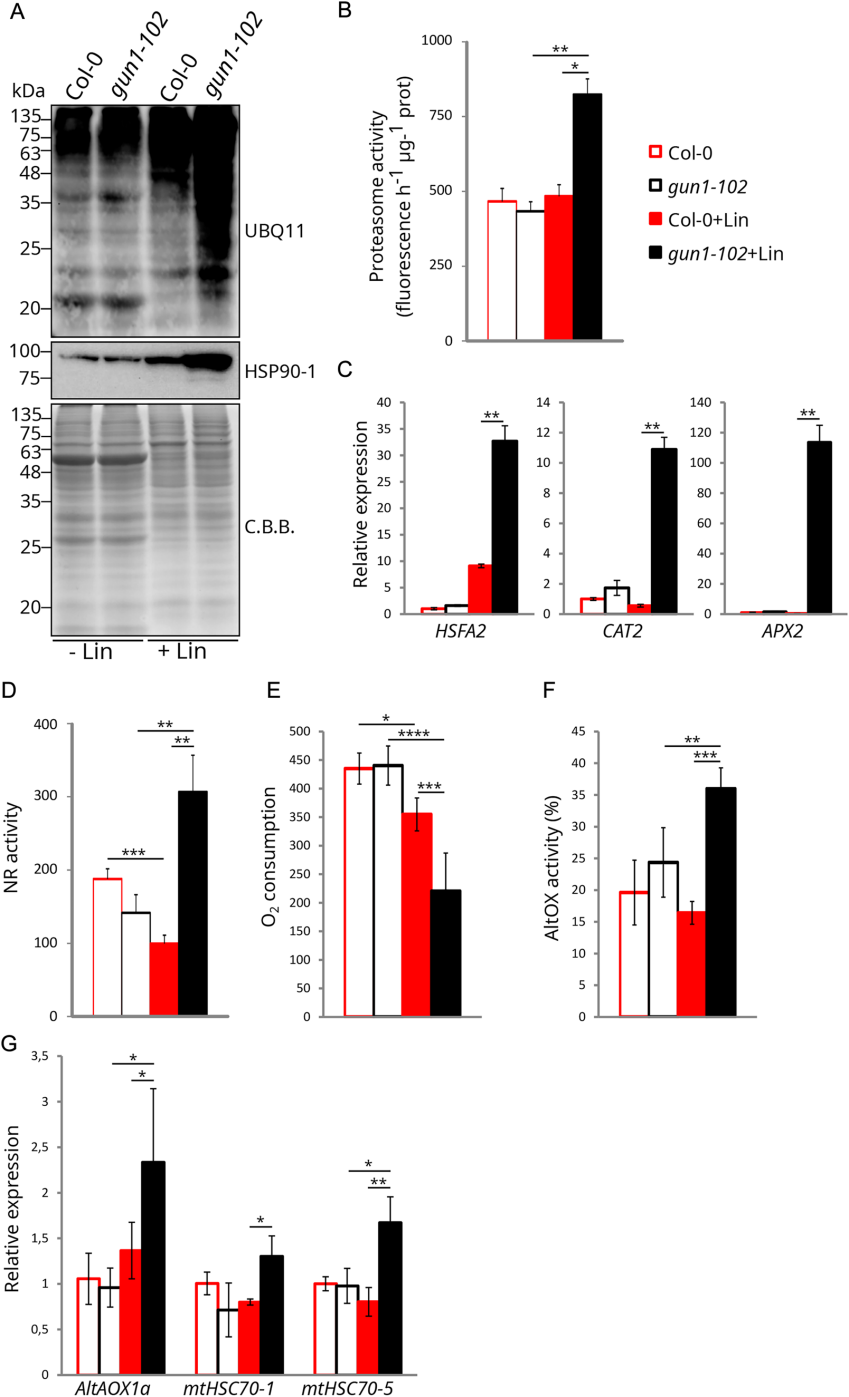
Validation of extra‐plastid protein hubs. (A) Cytosolic folding stress and ubiquitination in total protein extracts obtained from Col‐0 and *gun1‐102* samples grown in the absence or presence of Lin (±Lin). The immunoblots were obtained using antibodies specific for UBQ11 and cytosolic AtHSP90‐1, respectively. Coomassie Brilliant Blue (C.B.B.) staining of SDS–PAGE is shown as loading control. (B) Proteasome activity assay based on the level of fluorescence released by amino‐methyl‐coumarin. Asterisks indicate significant differences (*p < 0.05; **p < 0.01 according to ANOVA with Tukey's post hoc test). (C) Real‐time quantitative PCR of cytosolic ROS and the stress‐related marker genes *HSFA2*, *CAT2* and *APX2*. Asterisks indicate significant differences (*p < 0.05; **p < 0.01 according to Student's *t*‐test). (D) NR enzyme activity. Asterisks indicate significant differences (*p < 0.05; **p < 0.01; *** *p* < 0.001 according to ANOVA with Tukey's post hoc test). (E) Measurement of mitochondrial oxygen consumption (nmol O_2_/min/mg protein) in Col‐0 and *gun1‐102* ± Lin samples, together with (F) mitochondrial alternative oxidase (AltOX) activity. Asterisks indicate significant differences (*p < 0.05; **p < 0.01; ***p < 0.001; **** *p* < 0.0001 according to ANOVA with Tukey's post hoc test). (G) Real‐time quantitative PCR of *AltOX1a*, *mtHSC70‐1* and *mtHSC70‐5* transcripts, used as molecular marker genes for the investigation of mitochondrion‐to‐nucleus retrograde signalling and mtUPR. Asterisks indicate significant differences (*p < 0.05; **p < 0.01 according to Student's *t*‐test). [Color figure can be viewed at wileyonlinelibrary.com]

To clarify the key processes and players involved in each of the investigated conditions and validate the computational methods, a set of hub proteins identified through PPI and co‐expression networks were further investigated. First, the increase in cytosolic folding stress observed in *gun1* + Lin samples was considered. Proteins involved in ubiquitination processes were found to accumulate in both Col‐0 and *gun1* Lin‐treated samples, together with proteins involved in plastid proteolysis. DAPs included peptidases, proteases, proteasome subunits and ubiquitination‐related processes in +Lin samples (Figure 2, Supporting Information S4: Figure S4 and Supporting Information S13: Table S3). Moreover, PPI networks highlighted a central topological role for Ubiquitin Carboxyl‐terminal Hydrolase 3 (UCH3) protein in *gun1 *+ Lin samples (Figure 3A). Accordingly, UBQ11‐specific immunoblot analysis of total protein extracts obtained from 6 days after sowing (DAS) seedlings revealed a higher amount of ubiquitinated proteins in *gun1‐102* + Lin samples, with respect to Col‐0 + Lin, and compared to untreated seedlings (Figure 4A). Similarly, the cytosolic chaperones HSP90s accumulated to higher levels in *gun1‐102* + Lin seedlings, in agreement with previous studies (Wu, Meyer, Richter et al. 2019; Tadini, Peracchio, et al. 2020). These findings implied enhanced cytosolic 26S proteasome‐mediated protein degradation, which was confirmed by measuring the release of amino‐methyl‐coumarin from the fluorogenic substrate Suc‐LLYY‐NH‐AMC (Figure 4B). Compared to the other genotypes and conditions, proteasome activity was higher in *gun1‐102* + Lin samples, further indicating an increase in both cytosolic folding‐stress and proteolysis (Figure 2). The contribution of the cytosolic compartment was further assessed by testing the level of expression of *HSFA2* mRNA, which codes for a transcription factor with a key regulatory role in the activation of the cellular response to several different stresses (Nishizawa et al. 2006), and the expression of genes coding the catalase CAT2 and the ascorbate peroxidase APX2, which are active in the peroxisome and the cytosol, respectively (M. Ono, Isono, et al. 2021; Fryer et al. 2003; Anjum et al. 2016). In agreement with the higher levels of cytosolic folding‐stress observed under the *gun1* + Lin condition (Tadini, Peracchio, et al. 2020; Wu, Meyer, Richter et al. 2019), RT‐qPCR analyses indicated that *HSFA2* expression was induced by Lin in Col‐0 and, at a higher level, in *gun1‐102* plants (Figure 4C). *CAT2* and *APX2* gene expression was markedly upregulated in *gun1‐102* + Lin seedlings, whereas no induction was detected in Col‐0 + Lin.

Moreover, the enzyme NIA2, which is involved in nitrogen assimilation, was found to be a PPI hub in the *gun1* + Lin condition (Figure 3A), and its topological relevance is compatible with its nitrate reductase (NR) activity. Upon growth on Lin, NR activity decreased in Col‐0 seedlings, compared to untreated samples, while it was markedly increased in *gun1‐102* (Figure 4D). In addition to assimilating nitrate, NR can participate in the generation of nitric oxide (NO), which is known to induce the upregulation of the alternative oxidase (AltOX) under stress conditions (X. Huang et al. 2002; Salgado et al. 2013). The mitochondrial compartment appears to play a critical role in *gun1* + Lin samples, relative to the other conditions—as indicated by the presence of the GO categories ‘TCA cycle’ and ‘mitochondrion respiration’ in the PPI network and co‐expression analyses (Figure 3, Supporting Information S2: Figure S2 and Supporting Information S3: Figure S3). NO serves as a crucial signalling molecule, yet its reversible interaction with COX in mitochondria can lead to detrimental effects by inhibiting respiratory chain activity (Wulff et al. 2009; Millar and Day 1996). Based on the insights that point to a possible interaction between NR activity and mitochondria, the O_2_ consumption and the AltOX activity were measured (Figure 4E,F). Under control conditions, O_2_ consumption levels were similar between Col‐0 and *gun1‐102* seedlings. However, in the presence of Lin, respiration decreased in both genotypes, with a more pronounced reduction observed in *gun1‐102* seedlings. With regard to AltOX activity, no differences were detected between untreated Col‐0 and *gun1‐102* seedlings, and no changes were observed in Col‐0 + Lin, while AltOX activity increased in *gun1‐102* + Lin seedlings. Notably, several proteins from the copper amine oxidase family, which are involved in NO generation induced by H_2_O_2_, were also identified as hubs in the GUN1 + Lin PPI network (Figure 3; Wimalasekera et al. 2011). In agreement with these observations, the expression of *AltOX1a* was promoted by Lin in the *gun1‐102* background only, together with transcripts of nuclear genes, such as *mtHSC70‐1* and *mtHSC70‐5*, which code for mitochondrion‐located proteins that are involved at different levels in stress responses. This suggests that the mitochondrial adaptive response also requires mitochondria‐to‐nucleus retrograde communication (Figure 4G). This response appears to be triggered by the Lin‐specific inhibition of plastid protein synthesis, as no major alterations in the morphology of mTP‐YFP mitochondria were detected among the different genotypes by confocal fluorescence microscopy (Supporting Information S6: Figure S6). These findings suggest that when plastid function is impaired and GUN1‐mediated plastid‐to‐nucleus signalling is absent, mitochondria, together with vacuolar and cytosolic compartments, assume a key role in cell detoxification.

### 2.4 The Atypical Behaviour of the PsbO Protein

**Figure 5:**
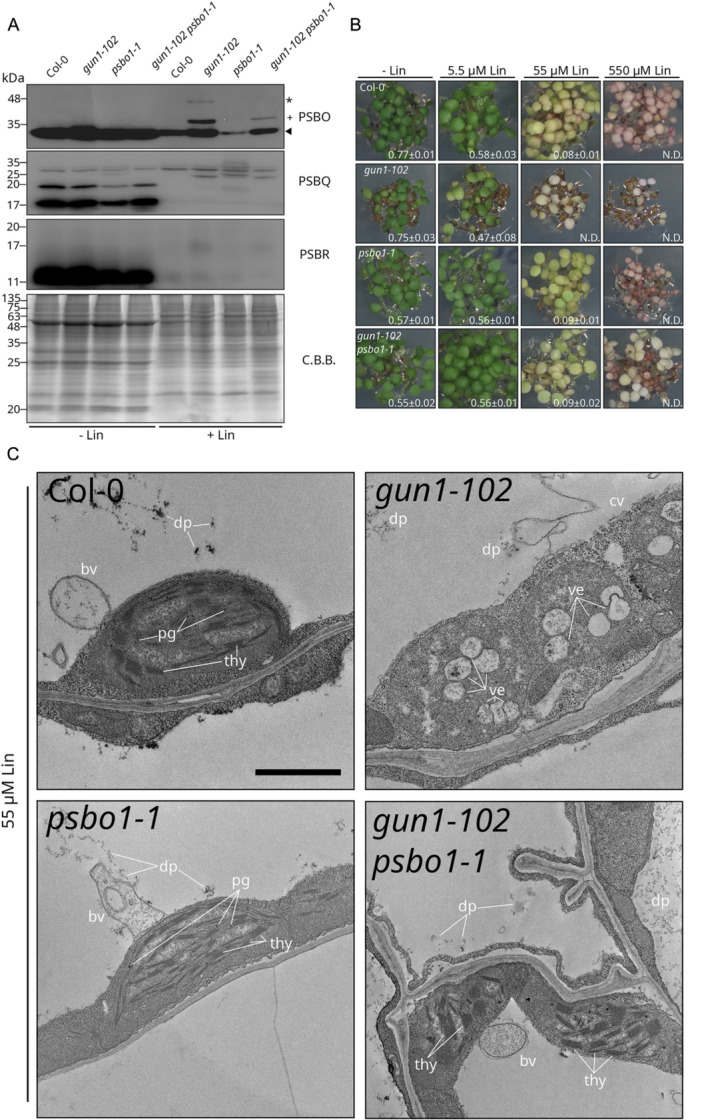
PsbO plays a role in the increased sensitivity of *gun1* seedlings to lincomycin. (A) Immunoblot analyses based on total protein extracts from seedlings grown in absence or presence of 550 µM Lin (±Lin). Extracts obtained from Col‐0, *gun1‐102*, *psbo1‐1* and *gun1‐102 psbo1‐1* genotypes were probed with antibodies specific for PsbO, PsbR and PsbQ. Coomassie Brilliant Blue (C.B.B.) staining of SDS–PAGE is shown as loading control. Note that the PsbO antibody reacts with both PsbO1 and PsbO2, and PsbO2 is detectable in the *psbo1* mutant genetic background. The arrowhead indicates the mature PsbO protein, the plus symbol (+) indicates the precursor protein and the band indicated by the asterisk (*) may refer to a post‐translational modification of PsbO precursor protein. (B) Visible phenotypes of 6 DAS wild‐type (Col‐0), single (*gun1‐102*, *psbo1‐1*) and double (*gun1‐102 psbo1‐1*) mutant seedlings grown on MS medium in the absence and presence of different concentrations of Lin (5.5, 55, 550 µM) in the growth medium. The *Fv/Fm* parameter (bottom right) is reported as an indicator of photosynthesis efficiency and the functional state of chloroplasts (average ± SD; *n* ≥ 15). (C) Transmission electron micrographs of mesophyll cells from 6 DAS seedlings of the indicated genotypes grown on MS medium with 55 µM Lin. thy, thylakoids; pg, plastoglobuli; ve, vesicle; bv, budding vesicles; dp, degradation products; cv, collapsing vacuole. The scale bar represents 1 µm. [Color figure can be viewed at wileyonlinelibrary.com]

Curiously, PsbO1 was the only plastid‐located co‐expression hub that was specific for *gun1* + Lin seedlings (Figure 3C and Supporting Information S15: Table S5). This observation is unexpected, since PbsO1 has so far been associated exclusively with the OEC, which is a component of Photosystem II. Furthermore, PsbO1 accumulates in plastids that are devoid of the photosynthetic apparatus, unlike most photosynthesis‐related proteins—including other subunits of OEC (PsbQ, PsbR; Figure 5A and Supporting Information S13: Table S3)—that have been found to be degraded as precursors in the cytosol or translationally inhibited (Wu, Meyer, Richter et al. 2019; Tadini, Peracchio, et al. 2020).

To further investigate the functional interaction between GUN1 and PsbO1 in the context of plastid perturbation, the *gun1‐102 psbo1‐1* double mutant was generated. Immunoblot analysis performed on total protein extracts from Col‐0, *gun1‐102*, *psbo1‐1* and *gun1‐102 psbo1‐1* seedlings showed that the PsbQ and PsbR subunits of the OEC did not accumulate in their mature forms in the presence of Lin (Figure 5A and Supporting Information S7: Figure S7). Conversely, PsbO1 and PsbO2 accumulate to relatively high levels in their mature forms upon exposure to Lin, particularly in *gun1‐102*‐containing genetic backgrounds (Figure 5A). PsbO signals were also detected in two distinct bands at higher molecular weight (see cross and asterisk) in *gun1‐102* + Lin samples, indicating the presence of precursor proteins that have retained their chloroplast and thylakoid Transit Peptides (cTP and tTP, respectively), as confirmed for both bands by mass spectrometric analyses (Supporting Information S16: Table S6). The lack of higher molecular weight bands in protein samples from purified plastids further supports the localization of these PsbO forms outside the plastid compartment (Supporting Information S7: Figure S7). Additionally, the shift in molecular weight of the uppermost band (asterisk) suggests that a fraction of PsbO pre‐proteins is subjected to post‐translational modification (Figure 5A and Supporting Information S7: Figure S7, see asterisk). Moreover, all transcripts that code for OEC subunits (PsbO1, PsbO2, PsbQ1, PsbQ2, PsbP1, PsbP2 and PsbR) behave like the *Photosynthesis‐Associated Nuclear Genes* (*PhANGs*) in *gun* mutants. Thus, expression of *PhANGs* was downregulated in both Col‐0 + Lin and *psbo1‐1* + Lin, while remaining relatively high in all genetic backgrounds containing the *gun1‐102* allele (Supporting Information S7: Figure S7). These findings indicate that, unlike other photosynthesis‐associated proteins encoded in the nucleus, PsbO1 and PsbO2 can accumulate as mature proteins in non‐green plastids, and therefore are not subjected to cytosolic degradation or post‐transcriptional inhibition.

Since *gun1* shows enhanced sensitivity to Lin (Tadini, Peracchio, et al. 2020; X. Zhao et al. 2018), the effects of the double *gun1‐102 psbo1‐1* mutant were tested at increasing Lin concentrations (Figure 5B). At 55 µM Lin, while Col‐0 showed a significant decrease in chlorophyll accumulation and PSII photosynthetic performance (0.08 ± 0.01), the *gun1‐102* mutant displayed no photosynthetic activity at all, and no chlorophyll. Moreover, *gun1* seedlings showed a significantly lower Maximum Quantum Yield of Photosystem II (*Fv/Fm*) value at 5.5 µM Lin (0.47 ± 0.08), thus corroborating the notion of a higher sensitivity to Lin, as reported previously (Tadini, Peracchio, et al. 2020; X. Zhao et al. 2018). On the other hand, the *psbo1‐1* mutant showed little or no change when grown in 5.5 µM Lin (0.56 ± 0.03) rather than under the control condition (0.57 ± 0.01). Strikingly, the introgression of the *psbo1‐1* mutation into the *gun1‐102* genetic background abolished the increased sensitivity to Lin—as shown by the Col‐0‐like *Fv/Fm* values and the visible phenotype of *gun1‐102 psbo1‐1* seedlings grown on medium containing 5.5 (0.56 ± 0.01) and 55 µM Lin (0.55 ± 0.02) (Figure 5B). On the other hand, in all genetic backgrounds, no photosynthetic activity at all was detected in the presence of 550 µM Lin. To further investigate at the level of plastid morphology, the partial restoration of *gun1‐102* phenotype upon introgression of the *psbo1‐1* mutation, 6 DAS Col‐0, *gun1‐102*, *psbo1‐1* and *gun1‐102 psbo1‐1* seedlings grown on medium with 55 µM Lin were observed through transmission electronic microscopy (TEM) (Figure 5C and Supporting Information S7: Figure S7). Col‐0 chloroplasts generally showed a lens‐shaped appearance with intact thylakoid membranes. However, as expected from their impaired photosynthetic parameters, alterations in chloroplast morphology were also observed. These included round‐shaped chloroplasts characterized by the presence of numerous plastoglobuli, together with membrane degradation products in the vacuole, and vesicles budding from the chloroplast envelope into the central vacuole (Figure 5C and Supporting Information S7: Figure S7). Conversely, in *gun1‐102* mesophyll cells, only a few thylakoid‐containing chloroplasts were observed, while most of the plastids showed no properly formed thylakoid membranes, together with large plastoglobuli and the accumulation of vesicles filled with electron‐dense material in the stroma (Figure 5C and Supporting Information S7: Figure S7). In a fraction of *gun1‐102* cells, the vacuolar membrane appeared to be disrupted, suggesting ongoing vacuole‐mediated cell death (Figure 5C). Intriguingly, *psbo1‐1* mesophyll cells contained fully developed chloroplasts with large amounts of thylakoid membranes and little or no sign of perturbation (Figure 5C and Supporting Information S7: Figure S7), relative to Col‐0, indicating further that plastids that are devoid of PsbO1 are less sensitive to chloroplast impairment and degradation when exposed to Lin. In accordance with this, the introgression of the *psbo1‐1* mutation into the *gun1‐102* genetic background led to a partial recovery of plastid/chloroplast morphology. In particular, *gun1‐102 psbo1‐1* mesophyll cells were largely characterized by chloroplasts with properly shaped thylakoid membranes, and no severely damaged or degraded chloroplasts were observed (Figure 5C and Supporting Information S7: Figure S7). Overall, these data indicate that PsbO—unlike any other photosynthesis‐related protein—accumulates in non‐photosynthetic plastids, and this trait correlates with chloroplast degradation upon impairment of plastid development.

### 2.5 PsbO Is Involved in Plastid Quality Control

**Figure 6:**
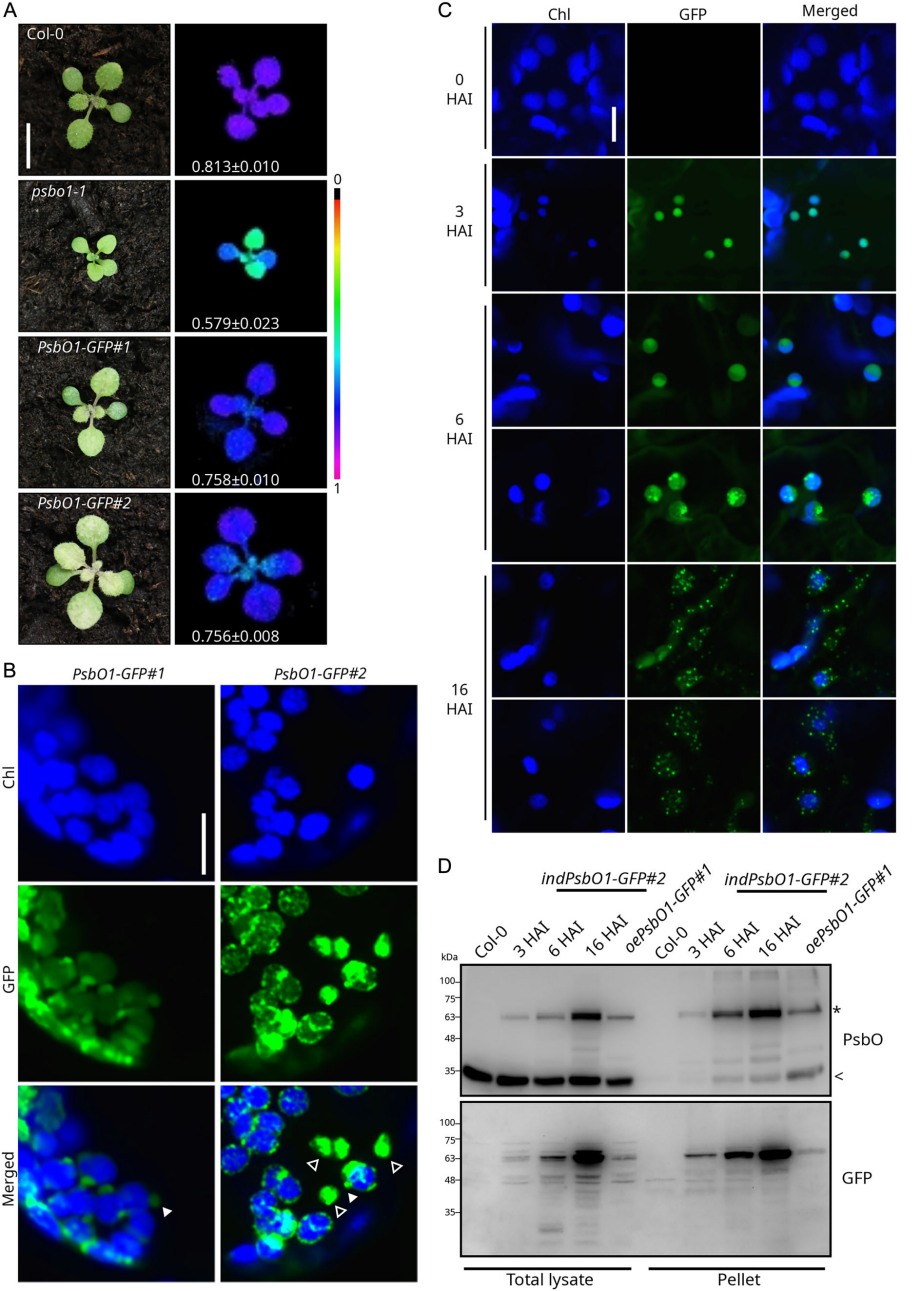
PsbO is involved in both chloroplast quality control and chloroplast degradation. (A) Visible phenotypes displayed by 12 DAS plantlets of the indicated genotypes and fluorometric data depicting levels of the *Fv/Fm* parameter (shown in false colours) and the average ± SD values (*n* ≥ 4). Scale bar: 1 cm. (B) Fluorescence signals from chlorophylls (Chl, blue) and GFP (green) detected in protoplasts obtained from leaves of the indicated genotypes were observed with a confocal microscope. Solid arrowheads indicate budding, vesicle‐like structures, while empty arrowheads show detached vesicles. Scale bar: 10 µm. (C) Fluorescence signals from chlorophylls (Chl, blue) and GFP (green) detected in mesophyll tissue obtained from the *indPsbO1‐GFP#2* line incubated for the indicated hours (HAI, hours after induction) in presence of DEX. Scale bar: 10 µm. (D) Immunoblot analyses of total lysates and pellet obtained by ultracentrifugation collected from leaves of the indicated genotypes and, where indicated, incubated in presence of DEX for the indicated hours. Filters were incubated with antibodies specific for PsbO and GFP. Asterisk (*) and arrowhead (<) indicate the GFP constructs and the endogenous PsbO, respectively. The comparable amount of endogenous PsbO in the total lysate indicates the equal loading in the ultracentrifuge tubes. [Color figure can be viewed at wileyonlinelibrary.com]

Reduced accumulation of the PsbO protein in the *gun1‐102 psbo1‐1* mutant background is accompanied by decreased sensitivity to Lin, as measured by PSII photosynthetic efficiency, and enhanced chloroplast integrity upon exposure to moderate (55 µM) levels of Lin (Figure 5). As PsbO proteins have been found to physically interact with chloroplast vesiculation (CV)—a protein that induces the disintegration of chloroplasts into discrete vesicles (S. Wang and Blumwald 2014), the role of PsbO1 as a hub in chloroplast quality control was investigated. To this end, two independent and viable *oePsbO1‐GFP* lines, driven by *CaMv35S* promoter, were generated and isolated. At 12 and 18 DAS, both lines showed a variegated phenotype, with slightly reduced *Fv/Fm* values relative to Col‐0 (Figure 6A and Supporting Information S8: Figure S8). A previously described *psbo1‐1* knock‐out line was shown to have a markedly lower *Fv/Fm* value and a reduced growth rate (Suorsa et al. 2016). Accumulation of the PsbO1‐GFP protein was verified by immunoblotting (Supporting Information S8: Figure S8), while the level of the endogenous PsbO protein was affected by the presence of the PsbO1‐GFP construct, which resulted in slightly reduced accumulation. The subcellular localization of PsbO‐GFP was analyzed in both lines by confocal microscopy observations of protoplasts obtained from well‐expanded leaves at 18 DAS. The GFP signal was observed as both dense foci and diffuse fluorescence within green plastids, co‐localizing with chlorophyll fluorescence. In addition, GFP was detected in vesicle‐like structures budding from chloroplasts and in vesicles that had become detached from plastids (Figure 6B). To clarify whether or not these vesicles were plastid‐derived, the *oePsbO1#1* line was crossed with a stable line ectopically expressing, under the control of *CaMv35S* promoter, an RFP‐tagged TIC20 subunit of the inner membrane translocon complex and analyzed by confocal microscopy. Large fluorescent foci of PsbO1‐GFP were mostly observed in vesicles that did not overlap with chlorophyll fluorescence and accumulated TIC20‐RFP, thus indicating that these structures were indeed derived from plastids (Supporting Information S9: Figure S9). To further investigate the dynamic of PsbO1‐GFP‐containing vesicle formation, *Arabidopsis* lines expressing the PsbO1‐GFP chimera under a dexamethasone (DEX)‐inducible promoter were generated. Two independent lines grown on MS medium supplemented with 4 µM DEX developed variegated leaf tissue, mimicking the phenotype of the *oePsbO1‐GFP* lines (Supporting Information S8: Figure S8). The kinetic of PsbO1‐GFP accumulation was monitored at 0, 3, 6 and 16 h after induction (HAI; Supporting Information S8: Figure S8). The localization of PsbO1‐GFP chimera over the course of DEX‐induction was observed through confocal microscopy (Figure 6C). At 3 HAI, chloroplasts exhibited diffused GFP fluorescence, with the signal distributed throughout the entire chloroplast, rather than being restricted to regions containing chlorophyll, as expected based on the typical PsbO accumulation pattern. By 6 HAI, the GFP signal became primarily localized to stromal regions, distinct from chlorophyll autofluorescence. In these chloroplasts, the signal appeared either diffusely distributed or condensed into distinct foci within the stroma, suggesting the formation of aggregation‐driven structures. At 16 HAI, the GFP signal was observed as condensed foci either within the chloroplasts or in vesicle‐like structures budding from them and extending toward the vacuole. These vesicles closely resembled those observed in *oePsbO1‐GFP* lines. The presence of GFP foci in both the *oePsbO1‐GFP* lines and in the inducible *indPsbO1‐GFP#2* line at 6–16 HAI ultimately supports the possibility that the PsbO1‐GFP chimera forms protein aggregates.

To test this hypothesis, total protein lysates from Col‐0, *oePsbO1‐GFP#1* and *indPsbO1‐GFP#2* leaves (treated with DEX for 3, 6, and 16 h) were subjected to ultracentrifugation and analyzed by immunoblotting to asses PsbO and GFP accumulation in the soluble and insoluble pellet fractions (Figure 6D). Notably, the insoluble fractions of *indPsbO1‐GFP#2* and *oePsbO1‐GFP#1* contained PsbO1‐GFP chimera signals (see asterisk), indicating the formation of insoluble aggregates. In contrast, endogenous PsbO (see arrowhead) was consistently detected in the total lysates of all samples but appeared in the insoluble pellet only when PsbO‐GFP accumulated. This suggests that PsbO1‐GFP aggregation may also influence the solubility of endogenous PsbO. Overall, these findings indicate that PsbO‐GFP accumulates in the stroma, disrupting chloroplast proteostasis (Jiang et al. 2017). This disruption leads to chloroplast swelling and, ultimately, the formation of aggregates that drive CV‐vesicle emergence (Figure 6C).

**Figure 7:**
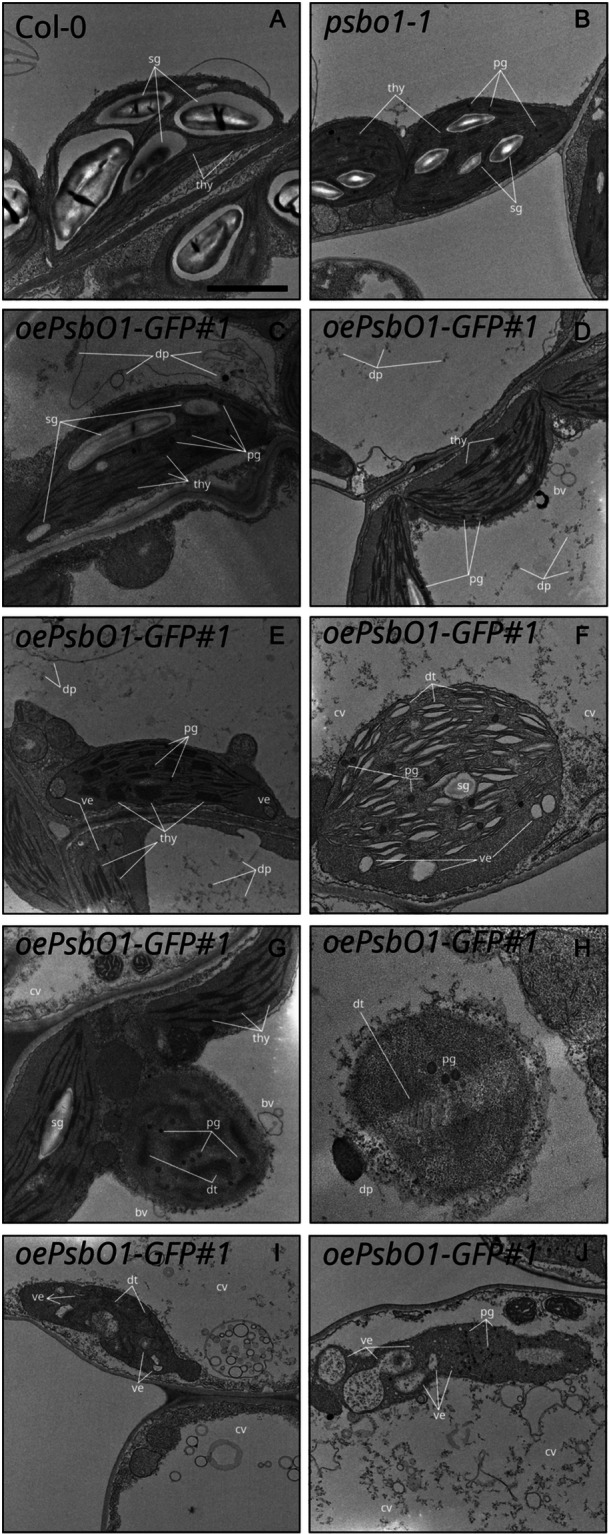
Transmission electron micrographs of mesophyll cells of Col‐0 (A), *psbo1‐1* (B) and *PsbO1‐GFP* (C–J) lines from 12 DAS plantlets grown on soil. thy, thylakoids; dt, degrading thylakoids; sg, starch granule; pg, plastoglobuli; ve, vesicles; bv, budding vesicles, dp, degradation products; cv, collapsing vacuole. Scale bar: 1 µm.

Since PsbO has been found in CV‐induced degradation vesicles (S. Wang and Blumwald 2014), the possibility that the ongoing disassembly of chloroplasts might be attributable to the PsbO1‐GFP protein was investigated by TEM analyses of leaves detached from plants grown on soil under physiological conditions (Figure 7). Col‐0 leaves displayed chloroplasts with thylakoid structures organized into grana stacks and stroma lamellae, together with large numbers of starch granules (Figure 7A), while *psbo1‐1* chloroplasts were characterized by swollen thylakoid structures, increased numbers of plastoglobuli and reduced amounts of starch granules (Figure 7B). These findings are compatible with the idea that the OEC plays a role in maintaining the structural integrity of thylakoid membranes (Suorsa and Aro 2007). On the other hand, chloroplasts obtained from *oePsbO1‐GFP* lines showed a wide range of alterations in plastid morphology, ranging from *psbo1*‐like chloroplasts (Figure 7C,D) to more markedly impaired plastids with severely damaged and degenerated thylakoid membranes (Figure 7E–G), to completely disintegrated chloroplast‐like organelles (Figure 7H–J). Closer inspection revealed structures associated with chloroplast disruption in the form of rounded chloroplasts that were detached from the plasma membrane and were clearly destined for the vacuole, as expected for a micro‐autophagy process (Figure 7G,H; see also Zhuang and Jiang 2019; Woodson 2022), or highly vesiculated chloroplasts compatible with fission‐type ATG‐ and PUB4‐dependent micro‐ and macro‐autophagy (Figure 7E,F,I,J; Jeran et al. 2021; Lemke et al. 2021; Woodson 2022; Tadini et al. 2023). In addition, vesicles similar to CV‐induced degrading vesicles, which formed in chloroplasts and migrated towards the central vacuole, were observed (Figure 7D,E; S. Wang and Blumwald 2014). Chloroplast degradation also led to the accumulation of electron‐dense material in the central vacuole, together with large amounts of membranes and degradation products (Figure 7C–J), like those previously observed in *gun1 ftsh2* and *gun1 ftsh5* genetic backgrounds (Tadini, Peracchio, et al. 2020). In several cells, chloroplast degradation was followed by vacuolar collapse and the disassembly of the entire cell structure (Figure 7G,I,J), similar to what has been described in ROS‐induced cell death (Woodson et al. 2015).

**Figure 8:**
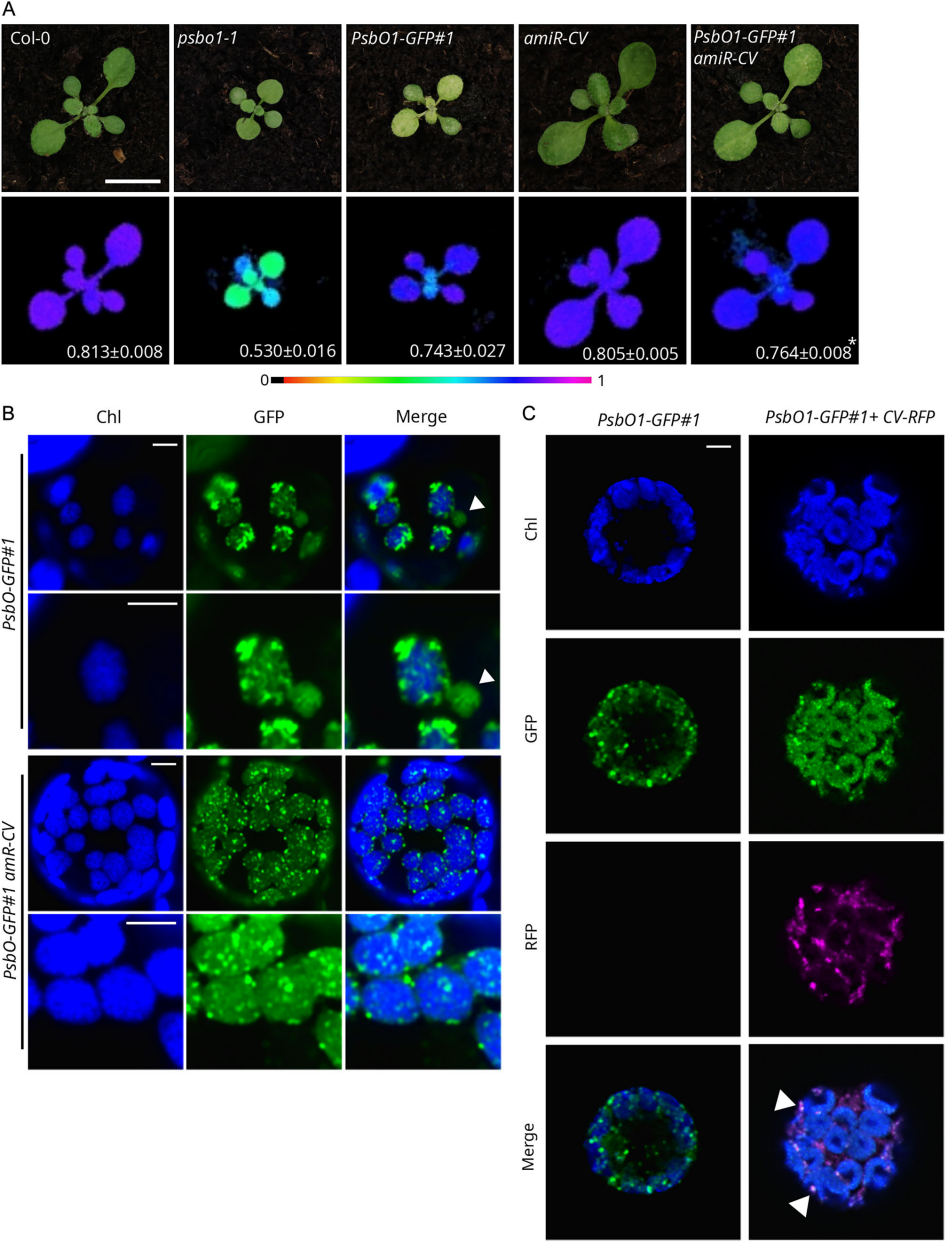
Functional interaction of PsbO with CV. (A) Visible phenotypes of 12 DAS plantlets of the indicated genotypes and fluorometric data representing the *Fv/Fm* parameter (in false colours) and the average ± SD values (*n* ≥ 4). Scale bar: 1 cm. (B) Fluorescence signals from chlorophylls (Chl, blue) and GFP (green) detected in protoplasts obtained from leaves of the indicated genotypes observed at different magnifications with a confocal microscope. Solid arrowheads indicate budding vesicle‐like structures. Scale bar: 5 µm. (C) Fluorescence signals from chlorophylls (Chl, blue), GFP (green) and RFP (magenta) detected in protoplasts obtained from the *PsbO1‐GFP#1* line and transformed with the *CV‐RFP* construct observed with a confocal microscope. Scale bar: 5 µm. [Color figure can be viewed at wileyonlinelibrary.com]

To ascertain whether the observed variegated phenotype displayed by the *PsbO1‐GFP* lines (Figure 6) was linked to CV activity, the *oePsbO1‐GFP#1* line was crossed with the *amiR‐CV* line, in which *CV* gene expression is silenced by an artificial microRNA (S. Wang and Blumwald 2014). Col‐0, *psbo1‐1*, *oePsbO1‐GFP#1*, *amiR‐CV* and the double mutant *oePsbO1‐GFP#1 amiR‐CV* were grown on soil under physiological conditions (Figure 8A). Strikingly, the resulting double‐mutant phenotype was partially rescued towards a more Col‐0‐like morphology. At 12 DAS, leaves showed minimal variegation and a slight improvement in photosynthetic parameters. By 18 DAS, the recovery of all parameters became even more pronounced (Supporting Information S10: Figure S10). The accumulation of the PsbO1‐GFP chimera was confirmed by immunoblot analyses using either anti‐PsbO or anti‐GFP antibodies (Supporting Information S10: Figure S10). In addition, confocal microscopy revealed that protoplasts isolated from mesophyll tissues of *PsbO1‐GFP#1 amiR‐CV* retained the GFP signal either as foci within chloroplasts or as diffuse patches that matched the chlorophyll autofluorescence (Figure 8B). The GFP signal observed in the *PsbO1‐GFP#1 amiR‐CV* double mutant was detected only within chloroplasts, whereas in the *oePsbO1‐GFP#1* line, foci were also detectable outside the plastids. These data support the idea that an imbalance of PsbO1 accumulation could act as a trigger for CV‐mediated chloroplast disassembly. To visualize such a functional interaction, protoplasts obtained from the *PsbO1‐GFP#1* line were transiently transformed with a construct expressing the *CV* coding sequence fused to *RFP* under the control of the *CaMV35S* promoter, and observed by confocal microscopy. As a control, *PsbO1‐GFP#1* protoplasts were analyzed as well. Strikingly, the RFP signal was found to diffuse into the cytoplasm and was observed in foci matching the GFP fluorescence, in agreement with the co‐localization of PsbO aggregates inside CV‐induced degradation vesicles (Figure 8C).

## 3 Discussion

Intracellular communication pathways rely on complex networks of molecular interactions that govern organelle development and play a key role in adaptation to the environment (Ng et al. 2014; Wu and Bock 2021). While GUN1 clearly plays a central role in transmitting information from developing and mature chloroplasts to the nucleus, which in turn alters nuclear gene expression, its primary functional role is still under debate (Koussevitzky et al. 2007; X. Zhao, Huang, et al. 2019; Tadini, Jeran, Pesaresi et al. 2020; Wu, Meyer, Richter et al. 2019; Tadini, Peracchio, et al. 2020; Colombo et al. 2016; Tang et al. 2024). In the present work, rather than focusing on the MF of GUN1 itself, we have described how the *Arabidopsis* seedling proteome reacts to the inhibition of plastid translation and chloroplast differentiation in a genetic context in which GUN1‐dependent plastid‐to‐nucleus signalling is lacking (Figure 1 and Supporting Information S1: Figure S1). Under these conditions, exposure to the antibiotic lincomycin leads to severe reduction in the size of the plastid proteome by (i) inhibiting translation in developing plastids, (ii) downregulating the expression of nucleus‐encoded plastid proteins and (iii) post‐transcriptionally suppressing their functions in the cytosol (Tadini, Jeran, Peracchio et al. 2020; Wu, Meyer, Wu, et al. 2019). Upon impairment of plastid translation, the production of most photosynthesis‐related proteins is inhibited, although a few plastid‐located subunits are unaffected, while a re‐orchestration of plastid housekeeping functions occurs (Figures 2, –, 4, Supporting Information S3: Figure S3 and Supporting Information S11: Table S1).

As we have shown here, plastid‐localized chaperones, proteases and ROS scavengers emerge as hubs under the Col‐0 + Lin condition, but not in the context of *gun1* + Lin (Figure 3). This finding indicates that, upon Lin treatment, plastid stress‐responses are mainly dependent on GUN1. In fact, the increase in accumulation of GUN1 protein upon impairment of plastid translation (Wu et al. 2018; Tadini, Jeran, Peracchio, et al. 2020) supports its role in plastid stress signalling as an integrator of multiple adaptive responses. Lin treatment has been shown to cause plastid‐protein aggregation and activation of the chloroplast UPR, which then triggers the nuclear response (Llamas et al. 2017). In turn, GUN1 physically and functionally interacts with the plastid–protein homoeostasis machinery, including key chaperones and proteases such as ClpC2 (a hub under the Col‐0 + Lin condition), cpHSP70, and Cpn60 (a hub in Col‐0/+Lin; Jia et al. 2019; Wu, Meyer, Richter et al. 2019; Tadini et al. 2016). In addition, GUN1 enhances the expression of plastid‐encoded NEP‐dependent housekeeping genes, including *Tic214*, *tRNAs*, *rRNAs*, ribosomal proteins and the protease subunit ClpP1, at both transcriptional and post‐transcriptional levels, thus further contributing to the maintenance of plastid homoeostasis (X. Zhao, Huang, et al. 2019; Tadini, Peracchio, et al. 2020; Tang et al. 2024). This also occurs under physiological conditions, as *gun1* seedlings occasionally show an albinotic phenotype (Ruckle et al. 2008; Tang et al. 2024). Indeed, albino/variegated phenotypes become more pronounced when *gun1* is combined with mutants that alter plastid–protein homoeostasis, or are subjected to heat, cold or chemicals (Lin and Norflurazon) (Tadini, Peracchio, et al. 2020; Marino et al. 2019; Lasorella et al. 2022; X. Zhao et al. 2018; Tadini, Jeran, Peracchio, et al. 2020; Tadini, Jeran, et al. 2020). These compensatory mechanisms, which are already active under physiological conditions, are enhanced by impairments of plastid function, probably owing to reduced plastid proteolysis capacity.

Increased cytosolic folding stress is observed in *gun1* seedlings upon Lin treatment as a result of the loss of GUN1‐mediated suppression of *PhANGs*, which in turn leads to the accumulation of precursor proteins in the cytosol (Tadini, Peracchio, et al. 2020). Thus, under the *gun1* + Lin condition, our analysis detected the cytosolic ubiquitin‐related protein UCH3 and the proteasome subunit PAF1 as hubs (Figure 3). In agreement with that finding, protein ubiquitination, together with the accumulation of chaperones and proteasome activity, were enhanced in *gun1* + Lin samples (Figure 4). Although our analysis detected clear plastid responses in Col‐0 seedlings upon Lin treatment, a whole‐cell re‐orchestration programme was triggered under this condition, as highlighted by enriched GO terms for nuclear activities, such as nuclear transport and RNA splicing and processing, Golgi vesiculation and mitochondrial reorganization (Supporting Information S2: Figure S2). Network topological analysis also revealed a number of vacuole‐related proteins that acquire hub status when Lin is applied to Col‐0 seedlings. This underlines the role of the vacuole in the clearance of protein aggregates and defective organelles, as also observed by electron microscopic analyses (Figures 3, –, 5 and Supporting Information S7: Figure S7; Muntz 2007; Woodson 2022; Shimada et al. 2018; Tan et al. 2019). Indeed, the vacuolar proteins SKD1 and VTI11, which are involved in the trafficking and delivery of protein cargos to lytic vacuoles (Muntz 2007; Sanmartín et al. 2007; Shahriari et al. 2010), were among the major topological players in the Col‐0 + Lin condition. Intriguingly, the RD21A protease, which is released from the vacuole during the programmed cell‐death cascade (Koh et al. 2016; Boex‐Fontvieille et al. 2015; Z. Wang et al. 2008), was elevated to hub status in the *gun1* + Lin condition, in place of the vacuolar proteins SKD1 and VTI11 observed in Col‐0 + Lin. It is therefore tempting to speculate that, when the normal GUN1‐mediated plastid response fails, the vacuole initiates autophagy and programmed cell death, as indeed is supported by the presence of highly disrupted plastids and collapsed vacuolar membranes in Lin‐treated *gun1* cells (Figure 5 and Supporting Information S7: Figure S7; Hatsugai et al. 2004). This also correlates with the altered redox state in *gun1* + Lin, as previously described (Fortunato et al. 2022), and is further highlighted by the presence of NIA2 and proteins belonging to the copper amine oxidase family (Wimalasekera et al. 2011) as PPI hubs in *gun1* + Lin samples (Figure 3), together with the more pronounced accumulation of cytosolic‐located ROS/RNS scavenging factors and increased NR activity (Figure 4). Intriguingly, levels of the HY5 transcription factor, a key regulator of photomorphogenesis and light‐responsive gene expression (L. Zhao, Peng, et al. 2019), also increased in response to Lin (Supporting Information S11: Table S1), yet it was identified as a PPI hub only under the *gun1* + Lin condition (Figure 3), possibly due to its role in mediating ROS accumulation, cell death and, importantly, *NIA2* expression (Gangappa and Botto 2016).

Mitochondria were also found to play a key role in the cellular events triggered by Lin. PPI functional modules and GO term analysis revealed enrichment in categories related to organelle organization and metabolism, which is specifically associated with mitochondrial functions (Figure 3, Supporting Information S2: Figure S2 and Supporting Information S3: Figure S3). Moreover, the activation of the mitochondrial AltOX and mitochondrial UPR gene expression was specific for the *gun1‐102* + Lin response (Figure 4). AltOX is a key marker of active mitochondrial retrograde signalling, and numerous studies have shown that it is upregulated when chloroplast function is impaired (Koussevitzky et al. 2007; Giraud et al. 2009). This highlights the compensatory role of the mitochondria in cellular adaptation when plastids fail to meet acute and/or ongoing challenges. Overall, we detected diverging behaviours of the extra‐plastid compartments in wild‐type and *gun1* seedlings upon Lin treatment. This indicates that, under stress, when plastid‐response coping mechanisms fail, cells resort to other compartments to address and cope with such challenges.

Following the comprehensive evaluation of proteome profiles, graph theory‐based computational strategies revealed that PsbO1 acts as a PPI hub in both genotypes under control conditions and is the only plastid‐located co‐expression hub that is detectable in *gun1* + Lin samples (Figure 3). PsbO, which is found in two copies in *A. thaliana*, is involved in linear electron transport across thylakoid membranes, as a subunit of the OEC, which is responsible for water splitting and P680 regeneration on the lumenal side of PSII (Allahverdiyeva et al. 2013; Yi et al. 2005). Almost all *PhANG*‐encoded proteins fail to accumulate in the presence of Lin, while the corresponding transcripts are suppressed by retrograde signalling pathways and a minor fraction is regulated at the post‐transcriptional level (Wu, Meyer, Wu, et al. 2019). When *PhANG* downregulation fails, protein accumulation is prevented by the inhibition of translation and degradation at the cytosolic level, although cytosolic precursor proteins can still be observed in *gun1‐102* + Lin samples (Figure 5 and Supporting Information S7: Figure S7; Tadini, Peracchio, et al. 2020; Wu, Meyer, Wu, et al. 2019; Jeran et al. 2021). Accumulation of PsbO was detected in plastids that were devoid of photosynthetic machinery and thylakoids—as in the case of the *Arabidopsis* mutants *tic56* and *ppi2*, and in seedlings treated with either Spectinomycin or Lin (Köhler et al. 2016). Intriguingly, PsbO accumulates as a mature protein in amounts that do not reflect the stoichiometry of the other OEC and PSII subunits (Figure 5 and Supporting Information S7: Figure S7). This in turn suggests that, under the *gun1‐102* + Lin condition, PsbO plays a role that is quite distinct from its OEC‐related functions. The unexpected accumulation of PsbO as the only *PhANG*‐encoded protein present in its mature form in Lin‐treated samples implies that it must make use of the non‐photosynthetic import machinery rather than the plastid‐encoded 1‐MDa TIC, since the latter's subunits *ycf1* and *ycf2* are not expressed in the presence of Lin (Supporting Information S11: Table S1).

Lack of GUN1 is correlated with increased levels of PsbO in plastids treated with Lin, and also results in higher sensitivity of seedlings to Lin (Figure 5). PsbO was identified in CV‐induced bodies and found to be involved in a PPI with CV itself (S. Wang and Blumwald 2014). Accordingly, the over‐accumulation of the PsbO1‐GFP chimera correlated with chloroplast degradation, and was partially reversed upon *CV* downregulation (Figures 6 and 7). Budding vesicles observed in *PsbO1‐GFP* chloroplasts strongly resemble micro‐ and macro‐autophagy processes (Jeran et al. 2021; Lemke and Woodson 2022; Lemke et al. 2021), while no vesicles were detected upon downregulation of *CV*. PsbO1‐GFP accumulation also resulted in formation of chimera aggregates, vacuolated structures and the disruption of vacuolar membranes that resemble those observed in cells that are undergoing programmed cell death (Woodson et al. 2015; Tadini et al. 2023). This correlates well with the identification of vacuole‐related hubs among *gun1* + Lin co‐expression hubs, including RD21A recently found to interact with PsbO in *Chlamydomonas* (van Midden et al. 2024; Koh et al. 2016). It has been suggested that the altered localization of PsbO‐GFP in the stromal compartment, not the over‐accumulation of PsbO itself, is the source of the variegated/chlorotic phenotype (Jiang et al. 2017). In this context, the simultaneous impairment of thylakoid biogenesis, together with the lack of downregulation of *PsbO* gene expression, would result in the accumulation of PsbO protein in the stromal compartment and, possibly, its aggregation, leading to enhanced chloroplast degradation under the *gun1* + Lin condition.

## 4 Conclusions

Using a holistic proteome analysis, we have provided new insights into the molecular mechanisms that are activated by the inhibition of chloroplast biogenesis in the presence/absence of the plastid‐to‐nucleus signalling pathway. Along with the description of proteomic rearrangements and the identification of particular proteins as network hubs, we provide evidence for adaptive responses triggered by aberrant plastid–protein localization. The accumulation of *PhANG*‐encoded proteins in the cytosol is known to activate a cytosolic folding‐stress response, which then triggers a feedback loop that inhibits translation and/or promotes pre‐protein degradation (Tadini, Peracchio, et al. 2020; Wu, Meyer, Wu, et al. 2019), followed by the activation of vacuole‐ and mitochondrion‐located compensation mechanisms. On the other hand, the abnormal localization of PsbO in the plastid stromal compartment appears to lead to its aggregation that could act as a signal for the degradation of altered plastids—with the aim of preventing ROS formation and oxidative damage. Overall, these data highlight the importance of plastid protein mislocalization as a source of adaptive responses to different stages of chloroplast development and altered environmental conditions, aimed at the repair or degradation of damaged plastids.

## 5 Materials and Methods

### 5.1 Plant Materials and Growth Conditions

Wild‐type *A. thaliana* (Col‐0) plants and mutants were grown on soil under controlled long‐day conditions (150 μmol m^−2^ s^−^^1^ 16 h/8 h light/dark cycles). The T‐DNA insertional mutants *gun1‐102, psbo1‐1* and *psbq1‐1 psbq2‐1 psbr‐1* have been described previously (Lundin et al. 2007; Tadini et al. 2016; Allahverdiyeva et al. 2013). *PsbO1‐GFP* lines were obtained by *Agrobacterium*‐mediated transformation of Col‐0 plants with an *Agrobacterium tumefaciens* strain that had been transformed with the plasmid pB2GW7 (gatewayvectors.vib.be), which carried the *PsbO1* coding sequence under the control of the CaMV35S promoter, cloned in‐frame with the *GFP* coding gene. The *amiR‐CV* line was previously described (S. Wang and Blumwald 2014). *PsbO1‐GFP amiR‐CV, PsbO1‐GFP TIC20‐RFP* and *gun1‐102 psbo1‐1* double mutants were obtained by manual crossing and segregation analysis. Inducible lines were obtained by cloning *PsbO1‐GFP* coding sequence in the DEX‐inducible *pOp/LhG4* system (Tadini et al. 2023) and by *Agrobacterium* transformation of *Arabidopsis* Col‐0 plants. For the analysis of mitochondrial morphology and dynamics, *gun1‐102* plants were crossed with *A. thaliana* Col‐0 lines expressing the yellow fluorescent protein (YFP) in the mitochondria, which were kindly provided by Prof. M. Z. For Lin treatment, seeds were surface‐sterilized and grown for 6 days (80 μmol m^−2^ s^−1^ on a 16 h/8 h dark/light cycle) on Murashige and Skoog medium (Duchefa) supplemented with 2% (w/v) sucrose, 1.5% (w/v) Phyto‐Agar (Duchefa) and Lin at 550 µM, unless otherwise indicated. For DEX treatment, Murashige and Skoog medium (Duchefa) was supplemented with 4 µM DEX. Leaf discs were vacuum infiltrated in a buffer containing 0.4% (w/v) Murashige and Skoog salts, 0.05% Tween‐20 and 4 µM DEX. Primers for mutant isolation and cloning are listed in Supporting Information S17: Table S7.

### 5.2 LC–MS/MS Analyses

For proteomic analysis, seedlings grown for 6 DAS on Murashige and Skoog medium supplemented with 2% (w/v) sucrose, 1.5% Phyto‐Agar and 550 µM Lin were homogenized in SDS lysis buffer [4% (w/v) SDS, 100 mM Tris/HCl pH 7.5, 100 mM DTT], incubated at 95°C for 5 min and centrifuged at 13 000*g* for 5 min. Crude protein extracts were digested with trypsin using two filter‐aided sample preparation (FASP) methods (Wiśniewski 2018). Prior to mass spectrometric analysis, peptides were dried in a Speed‐vac and desalted using Zip‐Tips (lC18; Millipore). Peptide samples (4 µg) were analyzed in a QExactive mass spectrometer coupled to a nano EasyLC 1000 (Thermo‐Fisher Scientific). The solvent compositions used were 0.1% (v/v) formic acid for channel A and 0.1% (v/v) formic acid, 99.9% (v/v) acetonitrile for channel B. Aliquots (4 µL) of each sample were loaded onto a custom‐made column (75 µm × 150 mm) packed with reverse‐phase C18 material (ReproSil‐Pur 120 C18‐AQ, 1.9 µm, Dr. Maisch GmbH), and eluted at a flow rate of 300 nL/min using a gradient from 2% to 35% (v/v) B for 80 min, followed by 47% (v/v) B for 4 min and 98% (v/v) B for 4 min. The mass spectrometer was operated in data‐dependent mode (DDA), and full‐scan MS spectra (300–1700 *m/z*) were acquired at a resolution of 70 000 at 200 *m/z* after accumulation to a target value of 3 000 000, followed by higher‐energy collision dissociation (HCD) fragmentation on the 12 most intense signals per cycle. HCD spectra were acquired at a resolution of 35 000, using a normalized collision energy of 25 and a maximum injection time of 120 ms. The automatic gain control (AGC) was set to 50 000 ions. Charge‐state screening was enabled, and single and unassigned charge states were rejected. Only precursors with an intensity above 8300 were selected for MS/MS (2% underfill ratio). Precursor masses previously selected for MS/MS measurement were excluded from further selection for 30 s, and the exclusion window was set at 10 ppm. Samples were acquired using internal‐lock mass calibration on *m/z* 371.1010 and 445.1200. For identification and characterisation of PsbO proteins, following western blot analysis, the corresponding protein bands were in‐gel digested with trypsin and analysed by quadrupole‐ion‐trap mass spectrometry as described previously (Domingo et al. 2023). PsbO proteins were identified by applying Turbo SEQUEST Bioworks 3.3 software (Thermo Electron Corporation, California, USA) to search the *A. thaliana* database (Araport11, www.arabidopsis.org/download, downloaded 1 December 2023) as described elsewhere (Domingo et al. 2023).

### 5.3 Raw Data Processing

Raw data, obtained from the literature or newly generated, were processed by the Sequest HT algorithm in Proteome Discoverer 2.1 software (Thermo‐Fisher Scientific). Experimental MS/MS spectra were compared with theoretical spectra obtained by in silico digestion of 39 345 protein sequences downloaded from UNIPROT in June 2020 (www.uniprot.org). The search criteria were set as: trypsin enzyme, three missed cleavages per peptide, mass tolerances of ±50 ppm for precursor ions and ±0.8 Da for fragment ions. The percolator node was used with a target‐decoy strategy to give a final false discovery rate (FDR) of ≤ 0.01 (strict) based on *q* values, considering a maximum deltaCN of 0.05. Only peptides with a minimum length of six amino acids, confidence at a ‘High’ level and Rank 1 were considered. Protein grouping and a strict parsimony principle were applied.

### 5.4 Proteomics Data Sets

Proteomics data used for this study were collected from three independent mass‐spectrometry experiments. The mass spectrometry data generated in this study (Data set 1, total protein content from Col‐0 ± Lin and *gun‐102* ± Lin, 6‐day‐old seedlings) has been deposited in the ProteomeXchange Consortium via the PRIDE (Perez‐Riverol et al. 2022) partner repository with the data set identifier PXD051970. Data set 2 originated from soluble proteins (Col‐0 ± Lin; *gun‐102* ± Lin, 6‐day‐old seedlings) and was published previously (Tadini, Peracchio, et al. 2020). Data set 3 originated from total protein contents (Col‐0 ± Lin; *gun‐101* ± Lin, 5‐day‐old seedlings) (Wu, Meyer, Richter et al. 2019).

### 5.5 Gene Ontologies in Protein Profiles

A preliminary functional evaluation of protein profiles was performed using the GO Term Enrichment for Plants tool in TAIR (Berardini et al. 2015) via the Panther (Mi et al. 2021) database. Using the protein profile of each biological replicate, enriched BPs and MFs terms were retrieved (*p* ≤ 0.05). Fisher's exact test and Bonferroni correction for multiple testing were set. Enriched BPs and MFs were compared by linear discriminant analysis (LDA) and those with an *F* ratio ≥ 5 and a *p* ≤ 0.01 were retained.

### 5.6 Label‐Free Quantitation Based on Spectral Counting

The spectral count (SpC) values of the identified proteins were normalized using a total‐signal normalization method, and compared using a label‐free quantification approach (Vigani et al. 2017). Specifically, data matrix dimensionality (*n* total = 36; *n* = 9/condition) was reduced by LDA (Sereni et al. 2018; Bari et al. 2023; Palma et al. 2021). As verifying normal distribution is not feasible with a small sample size (Branson and Freitas 2016), we proceeded under the assumptions of normally distributed and linearly separable data, with equal covariance matrices for each class. Pairwise comparisons (Col‐0 vs. Col‐0 + Lin; Col‐0 vs. *gun1*; *gun1* vs. *gun1* + Lin; Col‐0 + Lin vs. *gun1* + Lin) were performed and only proteins with an *F* ratio ≥ 5 and *p* ≤ 0.01 were considered. Fold change was estimated using the natural logarithm of the average spectral count (avSpC) ratio. The fold‐change value of proteins identified exclusively in one of the two compared conditions was set to ±5. Proteins selected by LDA were processed by principal component analysis and hierarchical clustering applying Ward's method and a Euclidean distance metric. Data processing was performed with JMP 15.1 SAS software.

### 5.7 Reconstruction of Differentially Expressed Functional PPI Network Modules

An *A. thaliana* PPI network model was reconstructed from the higher‐confidence DAPs (*n* = 326, *p* ≤ 0.001) based on comparisons between the characterized proteome profiles (Col‐0 vs. Col‐0 + Lin; Col‐0 vs. *gun1*; *gun1* vs. *gun1* + Lin; Col‐0 + Lin vs. *gun1* + Lin). To reconstruct the networks, the StringApp (Doncheva et al. 2019) for Cytoscape (Su et al. 2014) was used. PPIs were filtered by retaining exclusively those annotated in databases and/or experimentally determined with a score of ≥ 0.15. Proteins were grouped into functional modules based on BINGO 2.44 (Maere et al. 2005) using the following settings: *A. thaliana* (organism), hypergeometric test, Benjamini–Hochberg FDR correction, significance level ≤ 0.01. Reconstructed networks were visualized with Cytoscape. The colour code used for nodes indicates upregulated proteins in red and downregulated in light blue, based on avSpC normalization (SpC normalized in the range 0–100).

### 5.8 Protein–Protein Interaction and Co‐Expression Network Models

*Arabidopsis* PPI network models were reconstructed from all proteins identified under different conditions (Col‐0, *gun1*, Col‐0 + Lin, *gun1* + Lin). To reconstruct the networks, the StringApp (Doncheva et al. 2019) for Cytoscape (Su et al. 2014) was used. Retrieved PPIs were filtered by retaining exclusively those annotated in databases and/or experimentally determined, with a score of ≥ 0.6. According to these parameters, a fully connected network of 4669 nodes and 111 438 edges was built. Protein co‐expression network models were reconstructed by processing the *Arabidopsis* protein profiles for each condition (Col‐0, *gun1*, Col‐0 + Lin, *gun1* + Lin). To evaluate how correlations changed among the considered conditions, Spearman's rank correlation coefficient was computed only for proteins (*n* = 837) identified in all analyzed samples (*n* = 36, 9/condition); Spearman's rank correlation score ≥ |0.95| and a *p* ≤ 0.01 were set as thresholds. Correlation network models were processed with Cytoscape.

### 5.9 Topological Evaluation of PPI and Co‐Expression Network Models

Topological network analysis was performed with Cytoscape's plugin NetworkAnalyzer and Centiscape 2.2 (Assenov et al. 2008; Scardoni et al. 2015), as previously reported (Di Silvestre et al. 2021). Betweenness, centroid and bridging centralities were calculated for PPI models, while node degree was calculated for instances of co‐expression. For both models, nodes with centrality values above the average calculated for the whole network were considered to be hubs (Di Silvestre et al. 2018). The statistical significance of topological results was tested by considering randomized network models, which were reconstructed and analyzed with an in‐house R script based on VertexSort (to build random models), igraph (to compute centralities) and ggplot2 (to plot results) libraries.

### 5.10 Protein Sample Preparation and Immunoblot Analyses

Immunoblots were performed on total protein extracts. Fresh plant material was homogenized in sample buffer containing 20% (v/v) glycerol, 4% (w/v) SDS, 160 mm Tris‐HCl pH 6.8, 10% (v/v) 2‐mercaptoethanol (10 µL of buffer/mg of plant material; Tadini et al. 2023). Samples were incubated at 65°C for 15 min and centrifuged for 10 min at 16 000 *g* to pellet debris. Cleared homogenates were incubated for 5 min at 95°C. Samples (40 µL of protein, corresponding to 4 mg of fresh weight) were fractionated by SDS–PAGE [10% (w/v) polyacrylamide] (Schägger and von Jagow 1987) and then blotted onto polyvinylidene difluoride (PVDF) membranes (0.45‐µm pore size), and proteins of interest were detected with specific antibodies. Antibodies against PsbO (AS05 092) PsbQ (AS06 142‐16), PsbR (AS05 059), UBQ11 (AS08 307 A) and HSP90‐1 (AS08 346) were obtained from Agrisera (www.agrisera.com/), the GFP antibody (A‐6455) was obtained from Invitrogen (www.thermofisher.com) and anti‐RFP was purchased from Chromotek. Signal detection was performed with ChemiDoc Touch (Bio‐Rad; www.bio-rad.com) and Image Lab software (Bio‐Rad; www.bio-rad.com).

### 5.11 Isolation of Plastids From Lin‐Treated Samples

Plastids were isolated from 6 DAS seedlings grown in the presence of Lincomycin as described by Kunst (1998), with minor changes. In total, 150 mg of cotyledons were homogenized in 4 mL of a buffer containing 45 mM sorbitol, 20 mM Tricine‐KOH pH 8.4, 10 mM EDTA, 10 mM NaHCO_3_ and 0.1% (w/v) BSA fraction V, supplemented with cOmplete Proteinase Inhibitor Cocktail (Roche), filtered through Miracloth (Millipore) and centrifuged for 7 min at 700*g* at 4°C. The pellet was washed and gently resuspended in the same buffer. Finally, pellets were solubilized in 150 µL Laemmli buffer at 95°C for 5 min. Samples treated as described were immediately run in SDS–PAGE gels, blotted and immunodecorated with the indicated antibodies.

### 5.12 Isolation of Protein Aggregates

Separation of insoluble fractions from whole plant extracts was performed as previously described by Llamas et al. (2017) with minor changes. Liquid nitrogen frozen plant material was lysed using mortar and pestle in 4.5 mL in a buffer containing 30 mm HEPES‐KOH, pH 8.0, 60 mm KOAc, 10 mm MgOAc and 1% (v/v) glycerol, supplemented with cOmplete Protease Inhibitor Cocktail (Roche). Homogenates were filtered through Miracloth (Millipore), centrifuged at 3000 *g* for 10 min and supernatant was collected. Pellets were resuspended in 1 mL of supernatant and subjected to mechanical rupture through a syringe needle for ten times. Samples were then centrifuged at 3000 *g* for 10 min, the supernatant collected and combined with the previously stored supernatant and supplemented with 0.1% (v/v) Triton X‐100. After 10 min solubilization at 4°C, samples were centrifuged at 3000 *g* for 10 min. After collecting aliquots as total lysate input, the resulting supernatants were then subjected to centrifugation at 125 000 *g* for 1 h. The pellet was washed in the same buffer and centrifugated again at 125 000 *g* for 30 min. Finally, pellets were solubilized in 150 µL Laemmli buffer at 95°C for 5 min. Input samples were solubilized as well in equal volume of Laemmli buffer at 95°C for 5 min. Samples treated as described were immediately run in SDS–PAGE gels, blotted and immunodecorated with the indicated antibodies.

### 5.13 Transmission Electron Microscopy (TEM)

TEM analyses were performed as described previously (Jeran et al. 2021). Plant material was vacuum‐infiltrated for 4 h in a buffer containing 2.5% (v/v) glutaraldehyde and 100 mM sodium cacodylate, and incubated overnight at 4°C. Samples were post‐fixed in 100 mM cacodylate solution containing 1% (w/v) osmium tetroxide for 2 h at 4°C. Samples were then counterstained with 0.5% (w/v) uranyl acetate overnight at 4°C in the dark. Tissue dehydration was achieved by incubation for 10 min each in increasing concentrations of ethanol (70%, 80%, 90% v/v). Samples were incubated in 100% ethanol for 15 min and then in 100% propylene oxide for 15 min, twice. Epon‐Araldite resin was obtained by mixing Embed‐812, Araldite 502, dodecenylsuccinic anhydride (DDSA) and Epon Accelerator DMP‐30. Samples were infiltrated for 2 h with a 1:2 mixture of Epon‐Araldite and propylene oxide, then infiltrated for 1 h with a 1:1 mixture of Epon‐Araldite and propylene oxide, and finally incubated overnight at room temperature in a 2:1 mixture of Epon‐Araldite and propylene oxide. Samples were embedded in pure resin before polymerization at 60°C for 48 h. Ultra‐thin sections of 70 nm were cut with a diamond knife (Ultra 45°, DIATOME) and placed on copper grids (G300‐Cu, Electron Microscopy Sciences) prior to TEM (Talos L120C, Thermo Fisher Scientific) at 120 kV, and images were collected with a digital camera (Ceta CMOS Camera, Thermo‐Fisher Scientific).

### 5.14 Proteasome Activity

Proteasome activity was determined as reported previously (Paradiso et al. 2020). *Arabidopsis* seedlings (0.1 g) were ground in liquid nitrogen, homogenized in a 1:3 (w:v) ratio with extraction buffer (50 mM Hepes‐KOH, pH 7.2, 2 mM DTT, 2 mM ATP and 250 mM sucrose) and centrifuged at 20 000 *g* for 15 min at 4°C. Ten microlitres of supernatants (at 1 mg/mL protein concentration) were mixed with 220 μL of assay buffer (100 mM Hepes‐KOH, pH 7.8, 5 mM MgCl_2_, 10 mM KCl and 2 mM ATP). After 15 min of incubation at 30°C, the reaction was started by the addition of the fluorigenic substrate Suc‐LLYY‐NH‐AMC (Calbiochem), and the release of amino‐methyl‐coumarin (360 nm ex/460 nm em) was monitored over the course of 120 min using the Victor3 Multilabel Plate Reader (Perkin‐Elmer). Protein concentrations were measured with the Protein Assay System (Bio‐Rad) according to Bradford (1976), with serum albumin as standard. Statistical significance has been evaluated performing ANOVA with Tukey's post hoc test.

### 5.15 Nitrate Reductase (NR) Activity

NR (EC 1.7.1.1) activity was measured as described previously (Fortunato et al. 2019). *Arabidopsis* seedlings were ground in a mortar with 1:5 (w/v) extraction buffer (50 mM potassium phosphate pH 7.5; 1 mM EDTA, 1 mM DTT, 0,1 mM PMSF) and centrifuged at 20 000*g* for 15 min at 4°C. One volume of supernatants was incubated with five volumes of buffer (50 mM Hepes/KOH, pH 7.5, 0.04% (v/v) Triton X‐100, 2 mM EDTA, 10 μM Na_2_MoO_4_, 20 μM flavin adenine dinucleotide, 0.5 mM DTT, 20 μM leupeptin, 20 mM potassium nitrate). The reaction was started by the addition of 0.6 mM NADH. Aliquots (300 μL) were removed at 15‐min intervals, and the reaction was then stopped by adding 25 μL of 0.6 mM zinc acetate. Finally, 300 μL of 1% (w/v) sulphanilamide in 3 N HCl and 300 μL of 0.02% (w/v) *N*(1‐naphtyl)ethylendiamine dihydrochloride in 2.5% (v/v) H_3_PO_4_ were added, and the absorbance was read at 540 nm after 20 min. Statistical significance has been evaluated performing ANOVA with Tukey's post hoc test.

### 5.16 O_2_ Consumption and AltOX Activity Analysis

Mitochondrial activity was determined by monitoring oxygen consumption and AltOX activity. Measurements were performed using a test version of the NextGen‐O2k (Oroboros Instruments, Innsbruck) according to the methods developed previously (Vera‐Vives et al. 2022). Mitochondria were isolated from 6‐day‐old seedlings grown on Murashige and Skoog medium supplemented with 2% (w/v) sucrose and 1.5% (w/v) Phyto‐Agar, in the presence or absence of 550 µM Lin. Seedlings were homogenized at a 1:6 (m/v) ratio in 300 mM sucrose, 60 mM MES, 10 mM EDTA, 25 mM Na_4_P_2_O_7_, 10 mM KH_2_PO_4_, 1 mM glycine, 50 mM sodium ascorbate, 20 mM cysteine, 1% bovine serum albumin, and 1% polyvinylpyrrolidone (pH 8). Homogenates were filtered (50 µm membrane; Miracloth) and centrifuged at 2500 *g* for 5 min at 4°C. The supernatant was then centrifuged at 15 000 *g* for 15 min at 4°C. The resulting mitochondria‐enriched pellet was then resuspended in 200 µL of 300 mM sucrose, 10 mM MES, 2 mM EDTA, 10 mM KH_2_PO_4_ (pH 7.5). Respiration rates were normalized to total protein content, which was measured using an Infinite 200 PRO multimode plate reader (Tecan Group Ltd., Switzerland). The oxygen concentration was assessed in 2‐mL chambers at 22°C every 2 s, and samples were magnetically stirred at 750 rpm. Measurements were performed in the dark using 0.2 mg of total protein resuspended in respiration buffer (300 mM mannitol, 20 mM Hepes, 1 mM MgCl_2_, 20 mM KCl, 500 mM KH_2_PO_4_). Two measurements were done in parallel at each timepoint, taking advantage of the two chambers of the instrument. To stimulate respiration, malate (2 mM), pyruvate (5 mM) and thiamine pyrophosphate (TPP; 0.2 mM) were added, followed by ADP (1 mM). *n*‐propyl gallate (nPG; 0.5 mM) and Antimycin A (AA; 0.2 µM) were added to inhibit AltOX and Complex III activity, respectively. After each experiment, the respiration chambers were washed with 100% ethanol and rinsed with deionized water at least six times. AltOX capacity was calculated as the percentage of AA‐resistant respiration or nPG‐inhibited respiration relative to total respiration, normalized to leak respiration. Maximal capacity was normalized to the mitochondrial marker voltage‐dependent anion channel (VDAC). VDAC signals on western blots of crude extracts were used to normalize each value of maximal respiration. Data were collected from a minimum of six replicates across at least four independent experiments. Statistical significance has been evaluated performing ANOVA with Tukey's post hoc test.

### 5.17 Nucleic Acid Analyses

For real‐time quantitative PCR (RT‐qPCR) analyses, 1‐µg samples of total RNA were processed, using the iScript gDNA Clear cDNA Synthesis Kit (Bio‐Rad; www.bio-rad.com) for digestion of genomic DNA and first‐strand cDNA synthesis. RT‐qPCR analyses were performed on a CFX96 Real‐Time system (Bio‐Rad; www.bio-rad.com), using the primer pairs listed in Supporting Information S17: Table S7. *PP2AA3* (*AT1G13320*) expression served as the internal reference (Czechowski et al. 2005). Raw data were analyzed with Bio‐Rad CFX Maestro 1.1 v 4.1 software (Bio‐Rad; www.bio-rad.com). Samples were analyzed in triplicate. Statistical significance has been evaluated performing Student's *t*‐test.

### 5.18 Chlorophyll‐a Fluorescence Measurements and Chlorophyll Quantification

Chl‐a fluorescence was measured in vivo with an imaging Chl fluorometer (Walz Imaging PAM; walz.com), as previously described (Tadini et al. 2023). Dark‐adapted seedlings were used for measurements of the maximum quantum yield of PSII (*Fv*/*Fm*). The intensity of the blue measuring light and the saturating pulse intensity were both set to 4.

### 5.19 Protoplast Preparation and Confocal Microscopy

Protoplast isolation and transformation were performed as previously described (Yoo et al. 2007; Costa et al. 2012). Well‐expanded rosette leaves from 18 DAS were cut into strips of 1–5 mm with a scalpel blade. Leaf tissue was incubated in an enzyme solution containing 1.25% (w/v) cellulase [Onozuka R‐10 (Duchefa)] and 0.3% (w/v) Macerozyme R‐10 (Duchefa) for 3 h at room temperature in the dark. The suspension was filtered through a 50 μm nylon mesh and washed with a solution containing 154 mM NaCl, 125 mM CaCl_2_, 5 mM KCl and 2 mM MES (pH 5.7). For each protoplast transformation, 10‐μg aliquots of plasmid DNA were employed. Protoplasts or leaves were observed with an upright Nikon A1 confocal microscope using the following settings: GFP channel excitation 485 nm and detection 500–550 nm; RFP channel excitation 560 nm and detection 570–616 nm; chlorophyll channel excitation 485 nm and detection 663–738 nm.

## Supporting information

Figure S1.

Figure S2.

Figure S3.

Figure S4.

Figure S5.

Figure S6.

Figure S7.

Figure S8.

Figure S9.

Figure S10.

Table S1.

Table S2.

Table S3.

Table S4.

Table S5.

Table S6.

Table S7.

supmat.

## Data Availability

The mass spectrometry proteomics data generated in this study (Data set 1, total protein content from Col‐0 ± Lin and *gun‐102*±Lin 6‐day‐old seedlings) have been deposited to the ProteomeXchange Consortium (https://www.proteomexchange.org/) via the PRIDE partner repository with the data set identifier PXD051970.
