# Supplementary figures and images for: A Holistic Investigation of Arabidopsis Proteomes Altered in Chloroplast Biogenesis and Retrograde Signalling Identifies PsbO as a Key Regulator of Chloroplast Quality Control
Source: Plant Cell Environ. 2025 May 14;48(8):6373–96. doi: 10.1111/pce.15611 (PMC12223713; doi:10.1111/pce.15611)

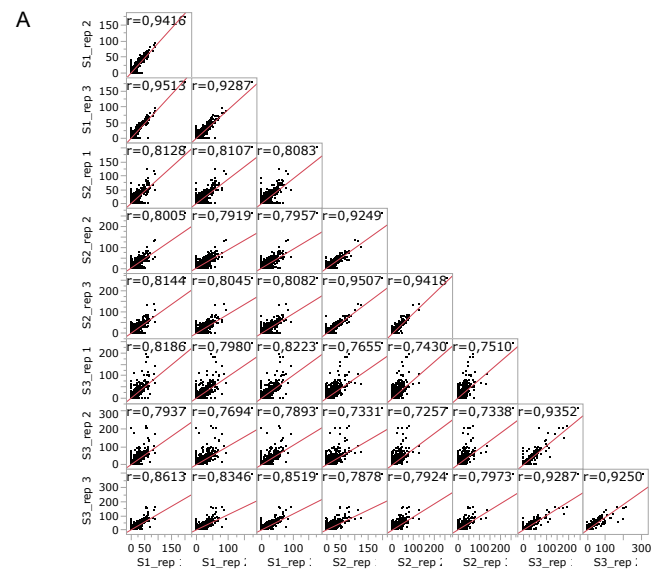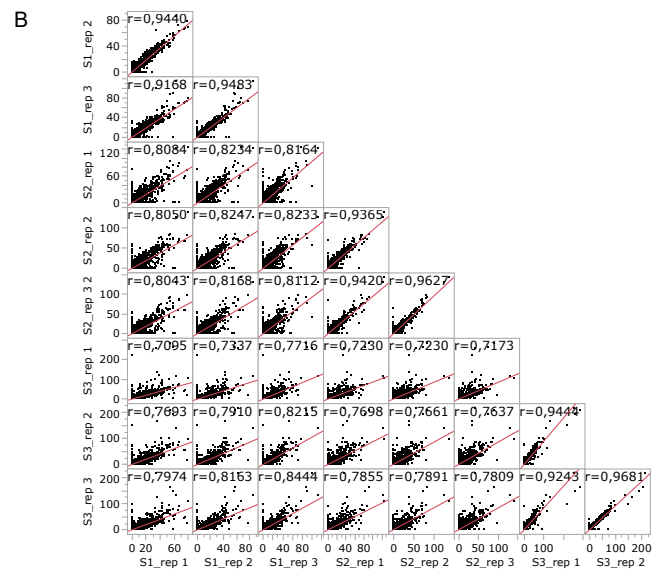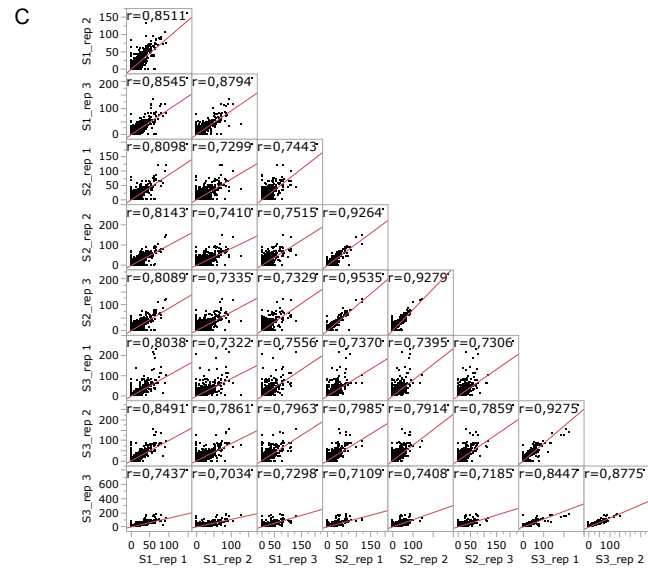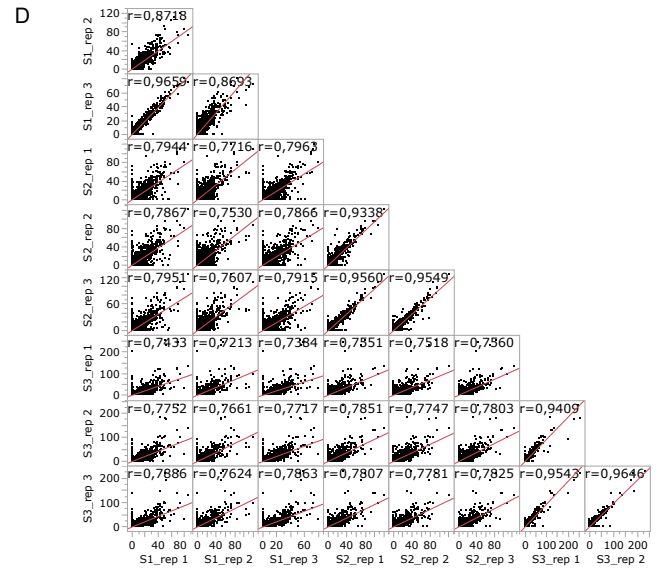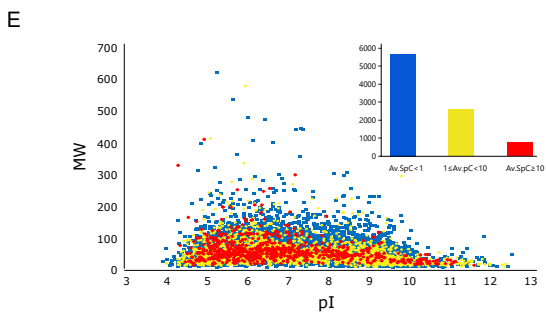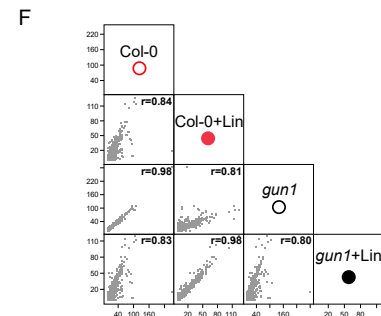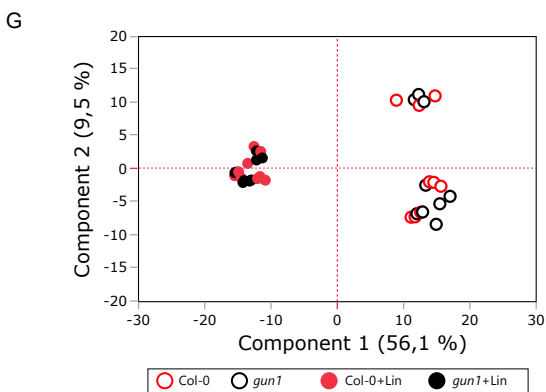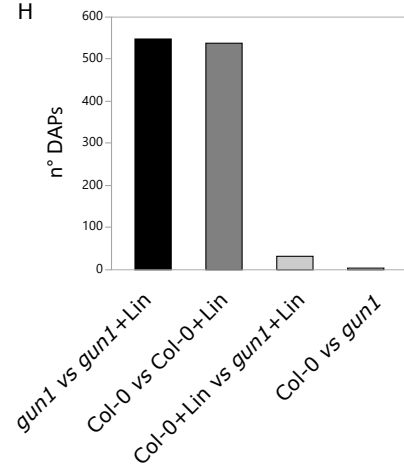

Supplement: Supplementary file 1 — Figure S1. [file PCE-48-6373-s003.pdf]

A

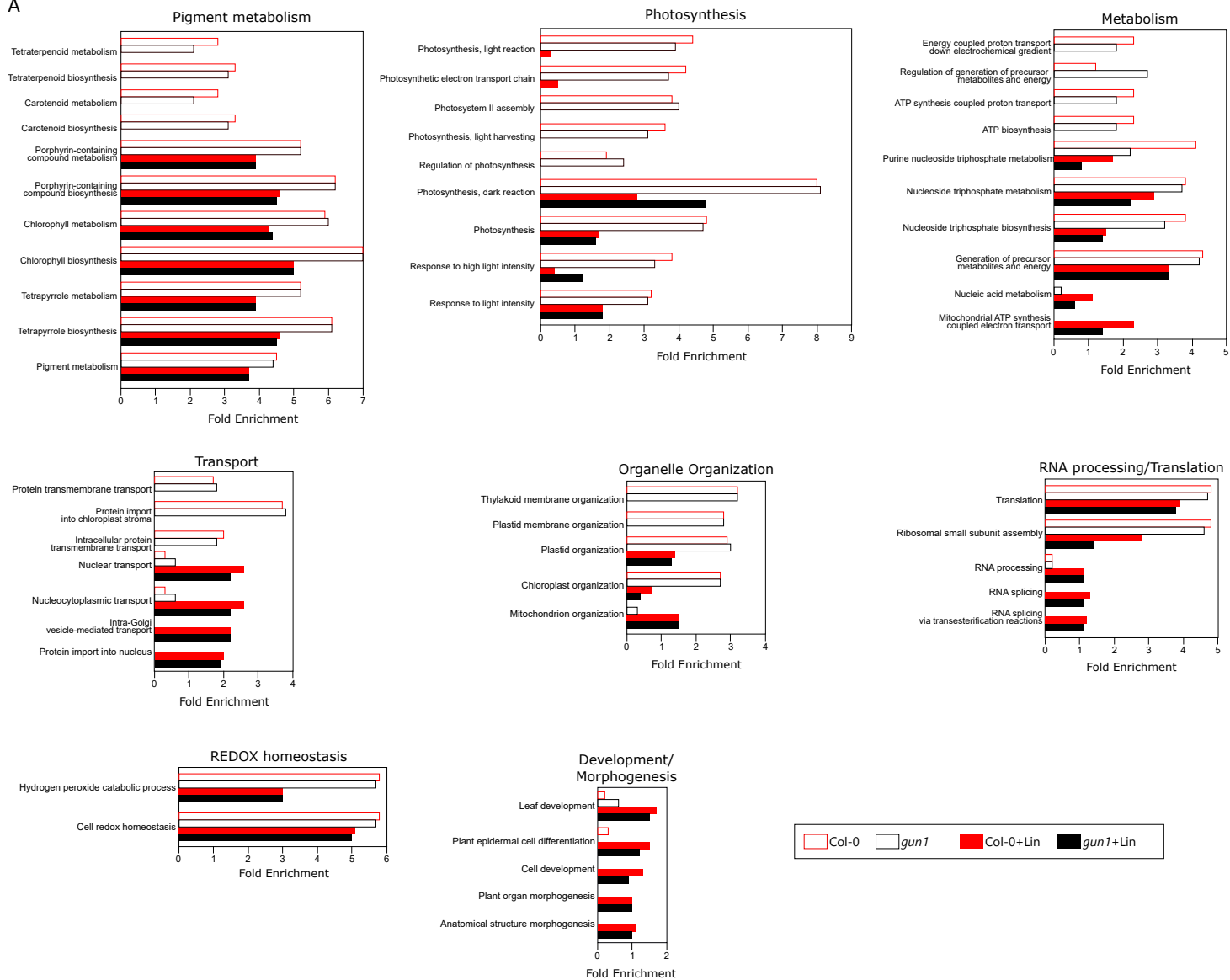

B

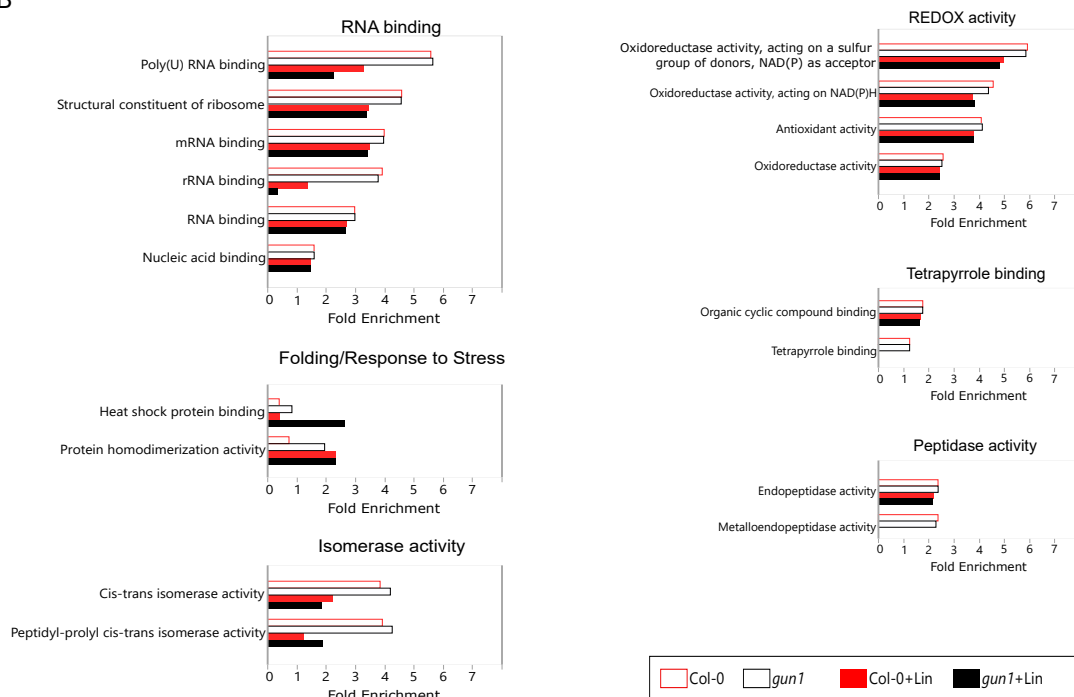

Supplement: Supplementary file 2 — Figure S2. [file PCE-48-6373-s009.pdf]

A

Col-0

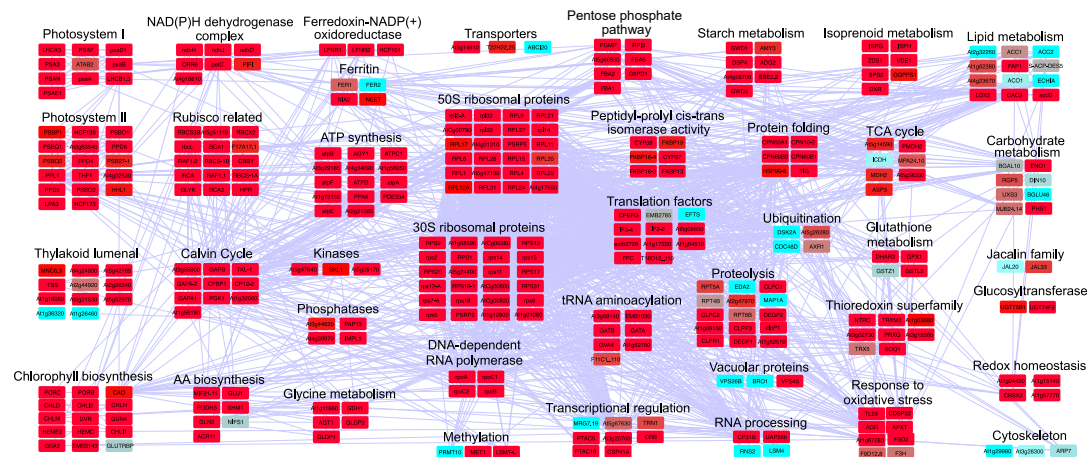

B

gun1

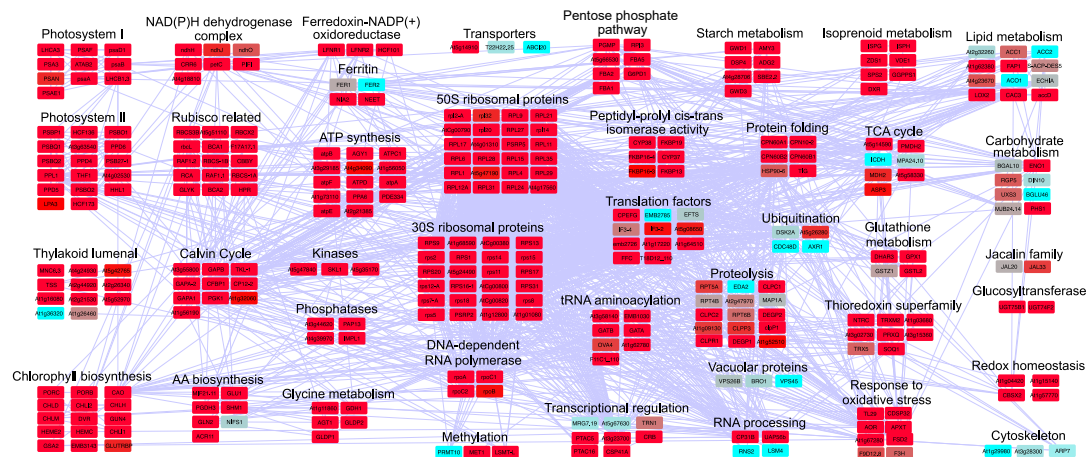

C

Col-0+Lin

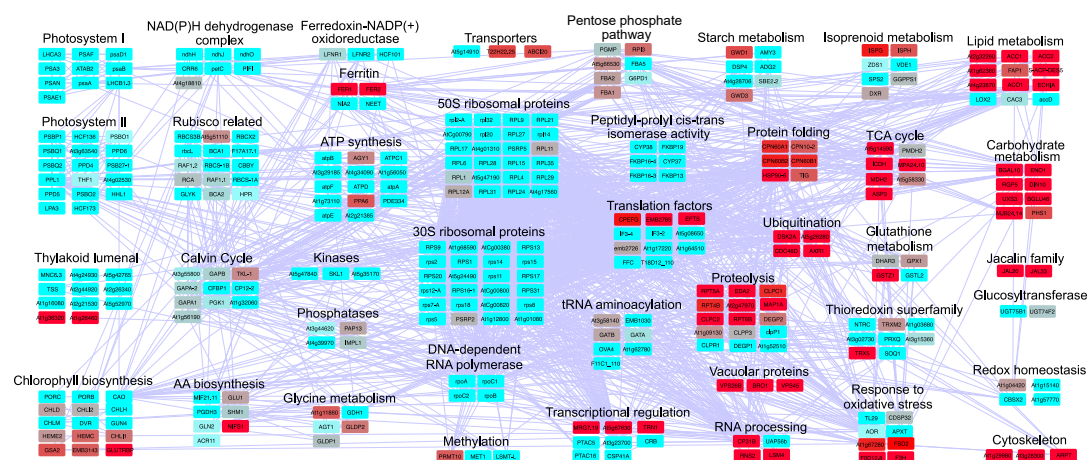

D

gun1+Lin

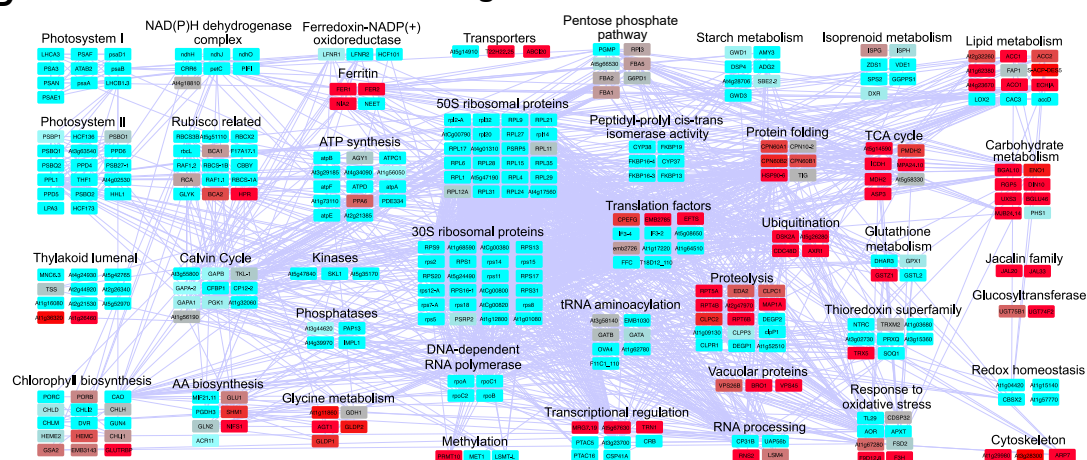

nSpC (0-100)

(Down-Regulated)---25

75---(Up-Regulated)

Supplement: Supplementary file 3 — Figure S3. [file PCE-48-6373-s017.pdf]

# PROTEOLYSIS

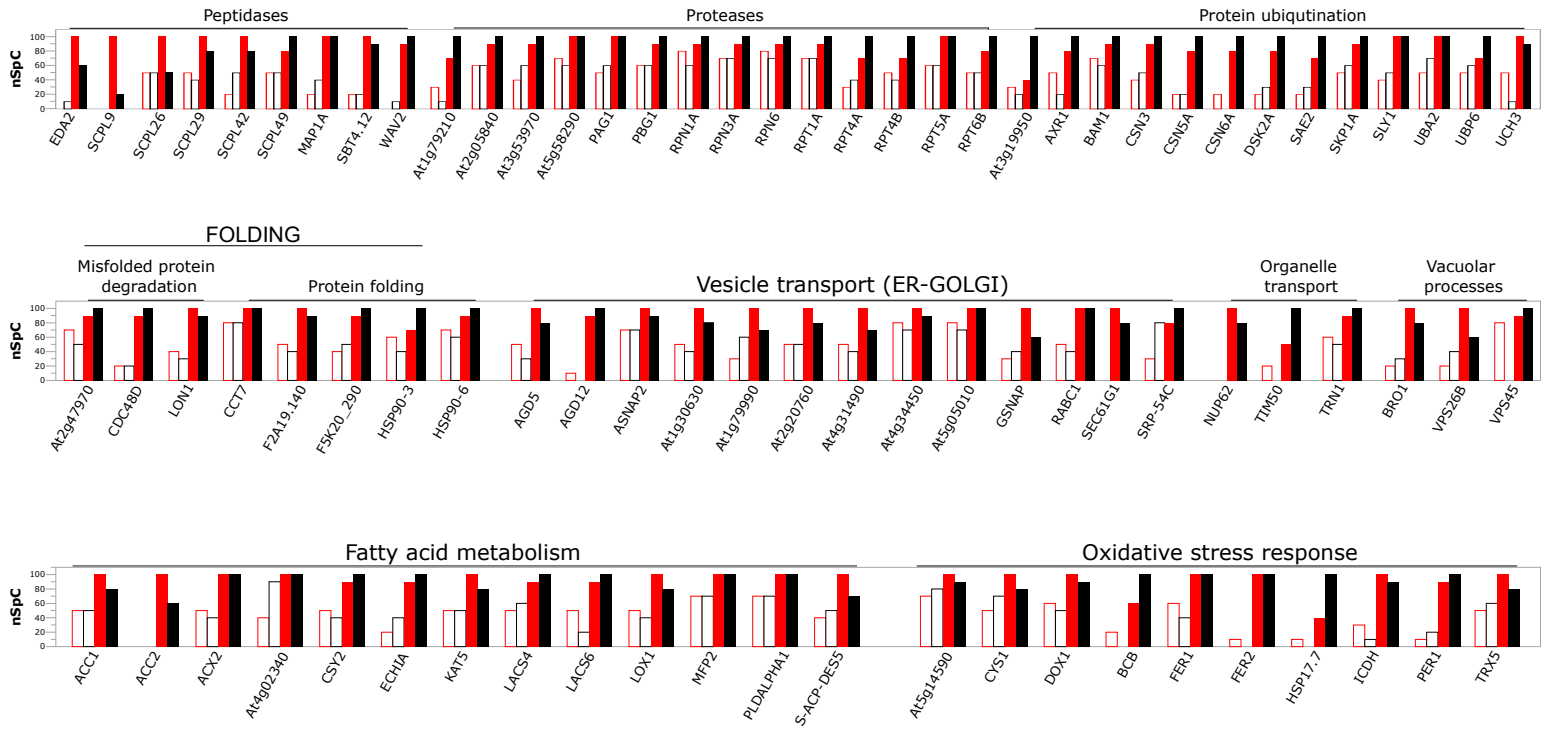

Supplement: Supplementary file 4 — Figure S4. [file PCE-48-6373-s015.pdf]

A

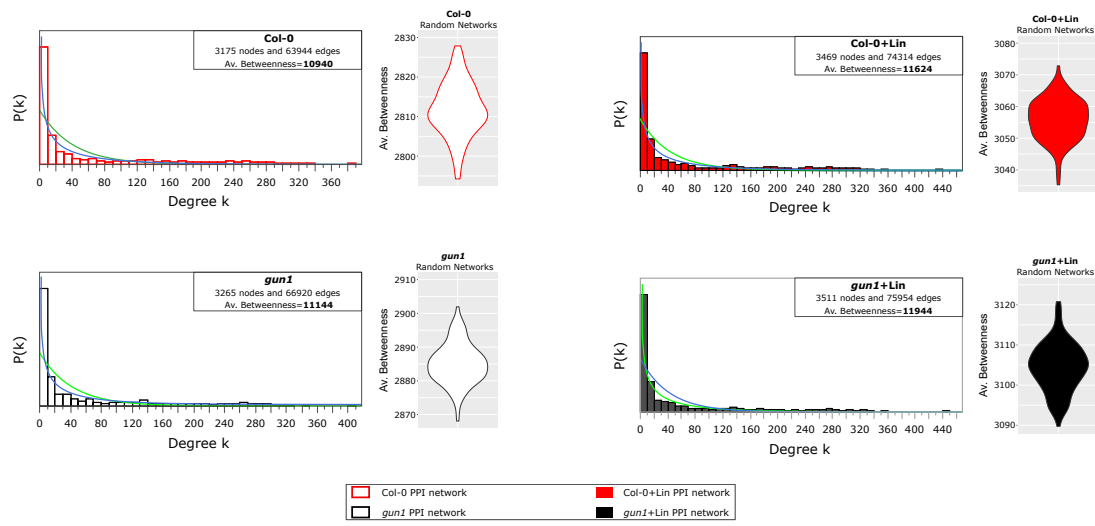

B

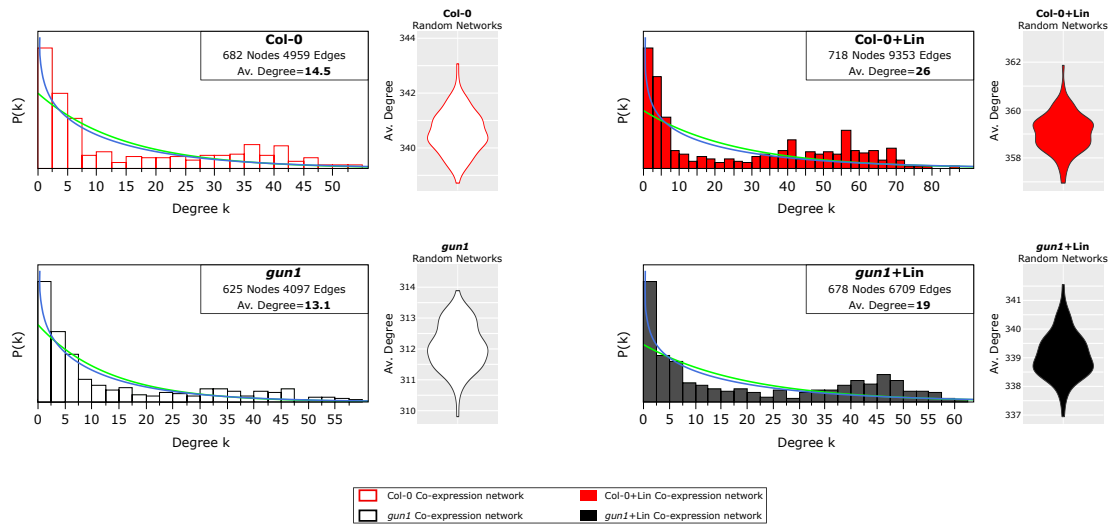

Supplement: Supplementary file 5 — Figure S5. [file PCE-48-6373-s005.pdf]

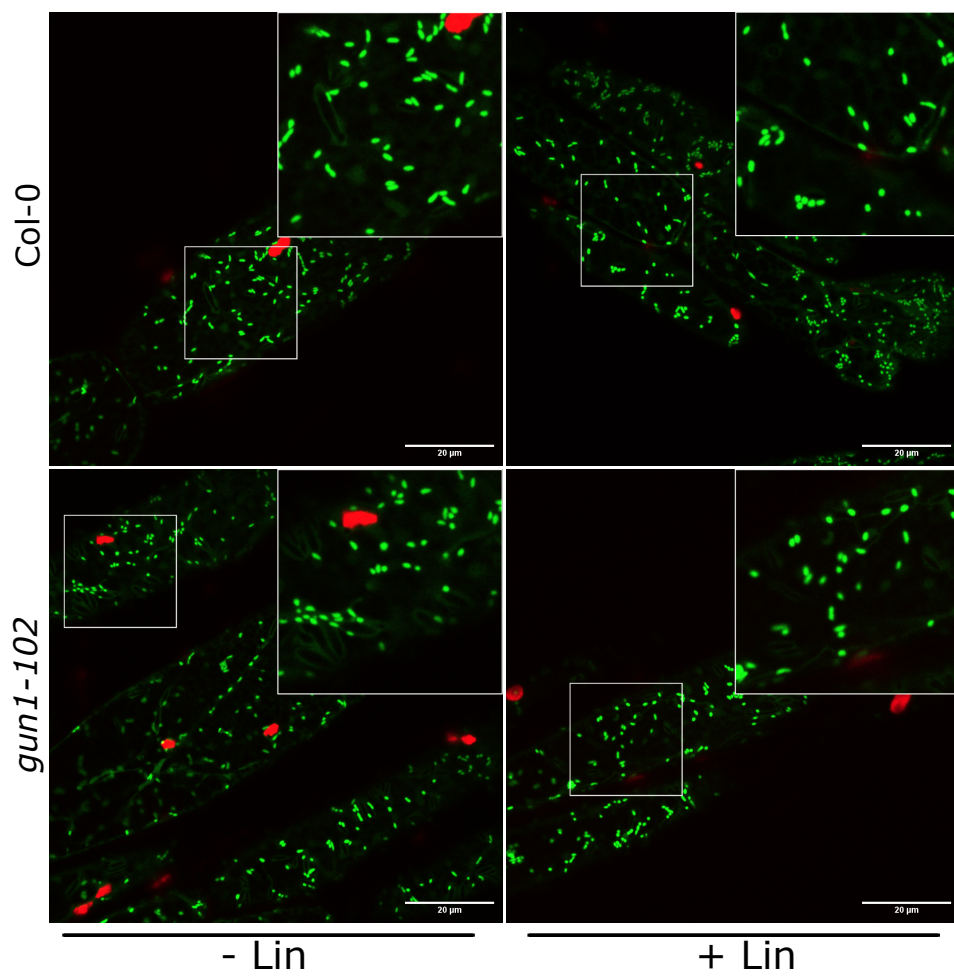

Supplement: Supplementary file 6 — Figure S6. [file PCE-48-6373-s001.pdf]

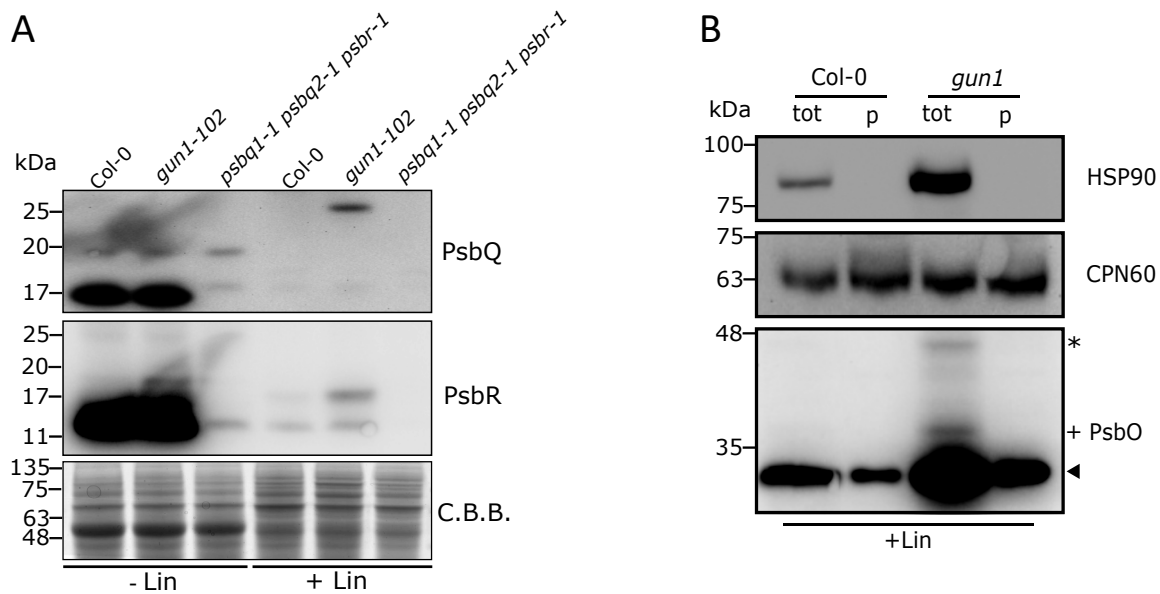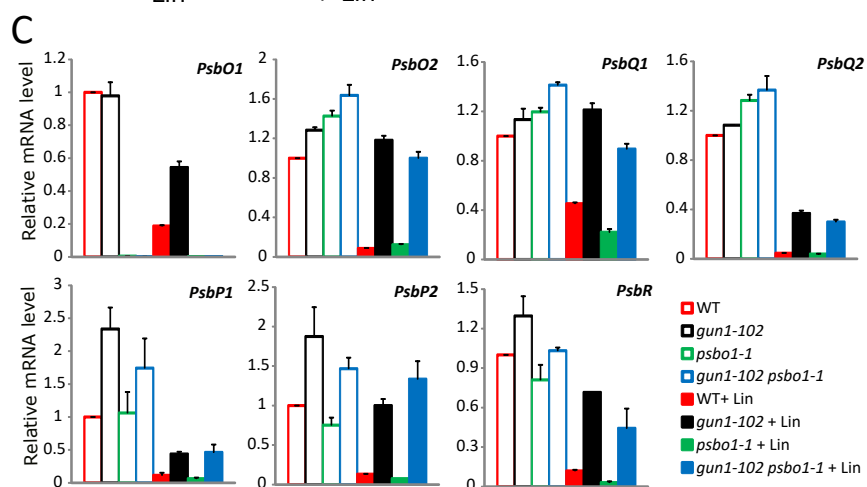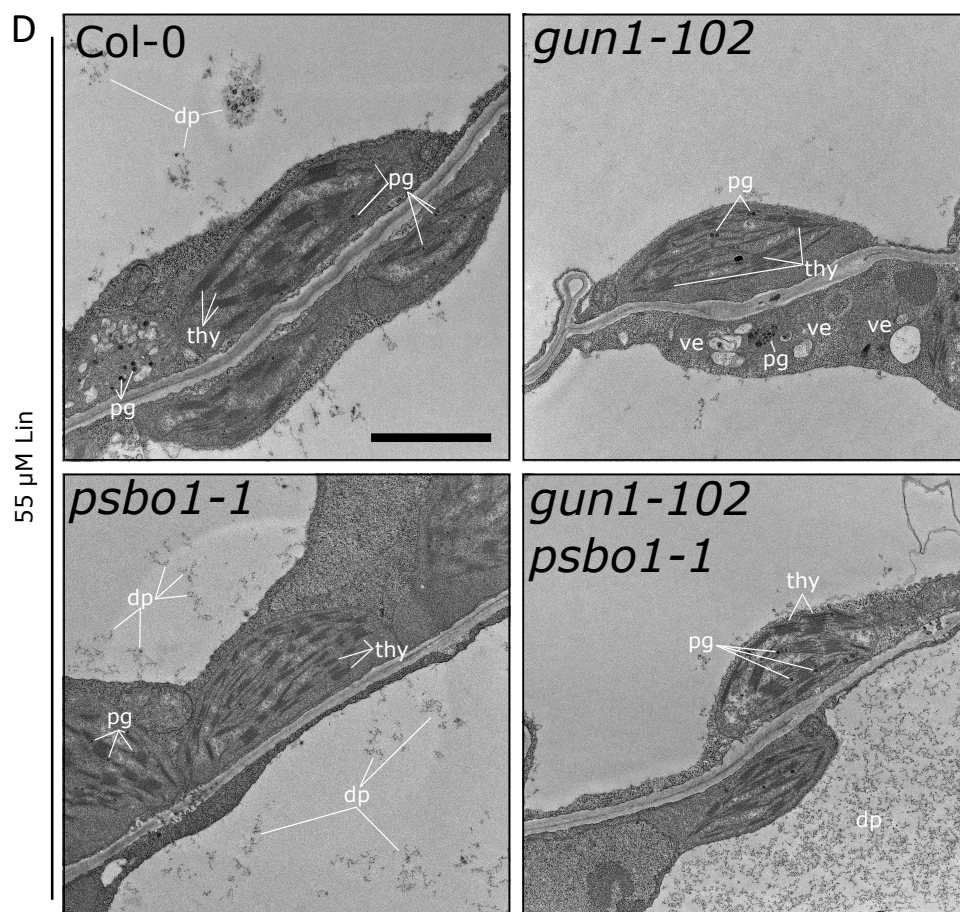

Supplement: Supplementary file 7 — Figure S7. [file PCE-48-6373-s011.pdf]

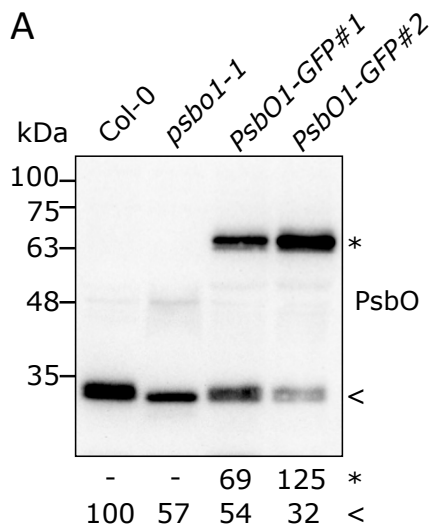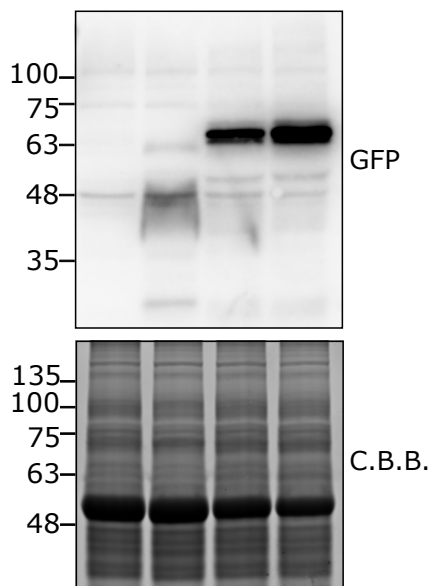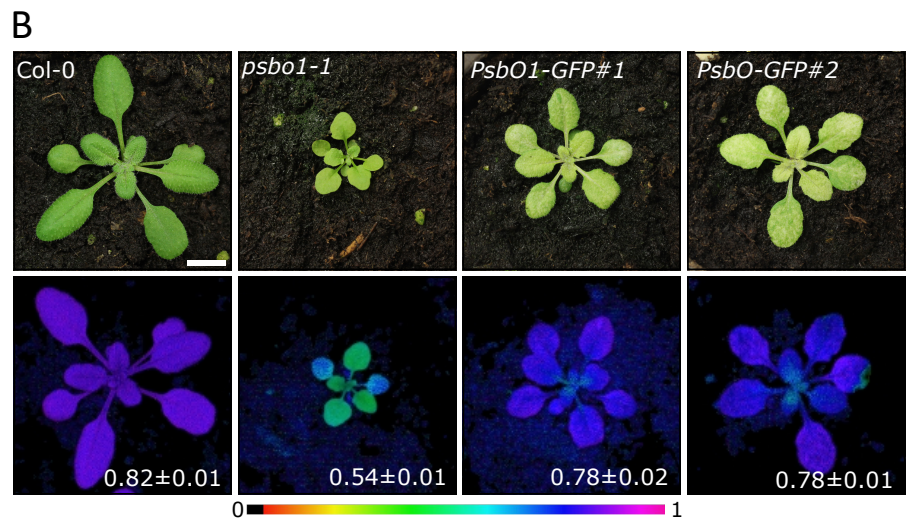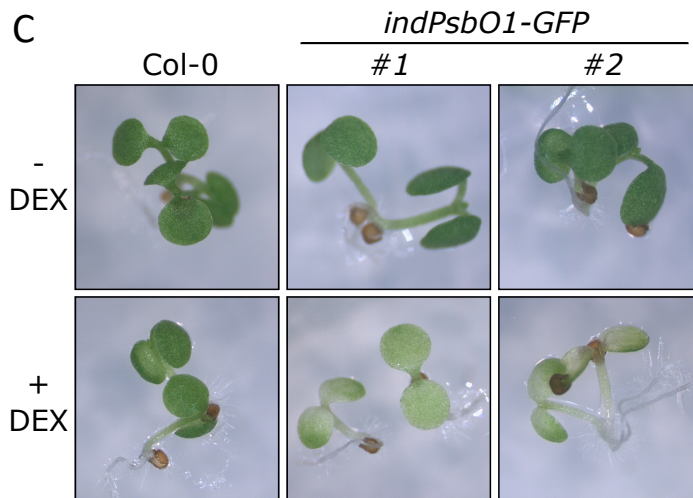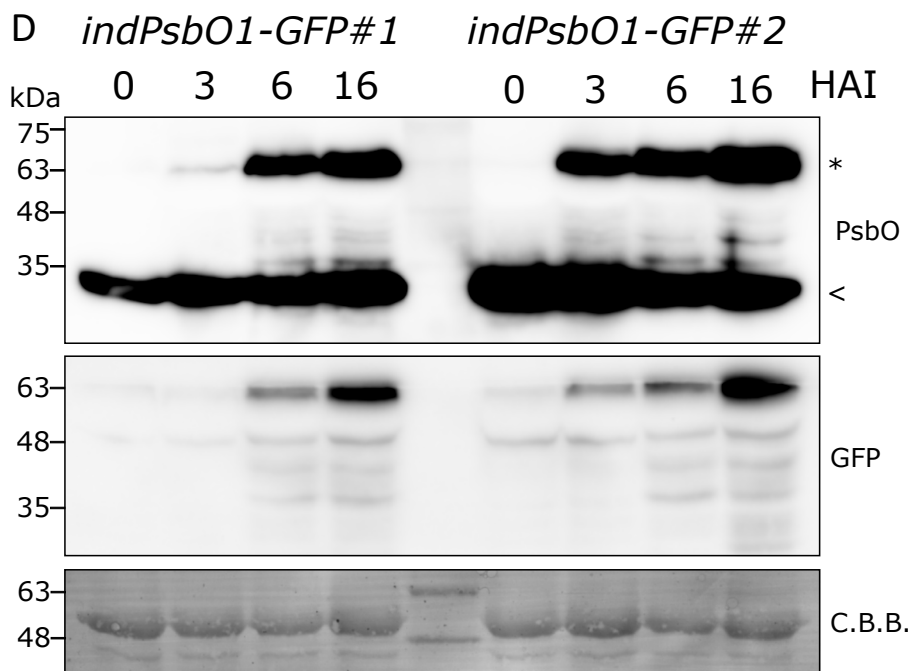

Supplement: Supplementary file 8 — Figure S8. [file PCE-48-6373-s006.pdf]

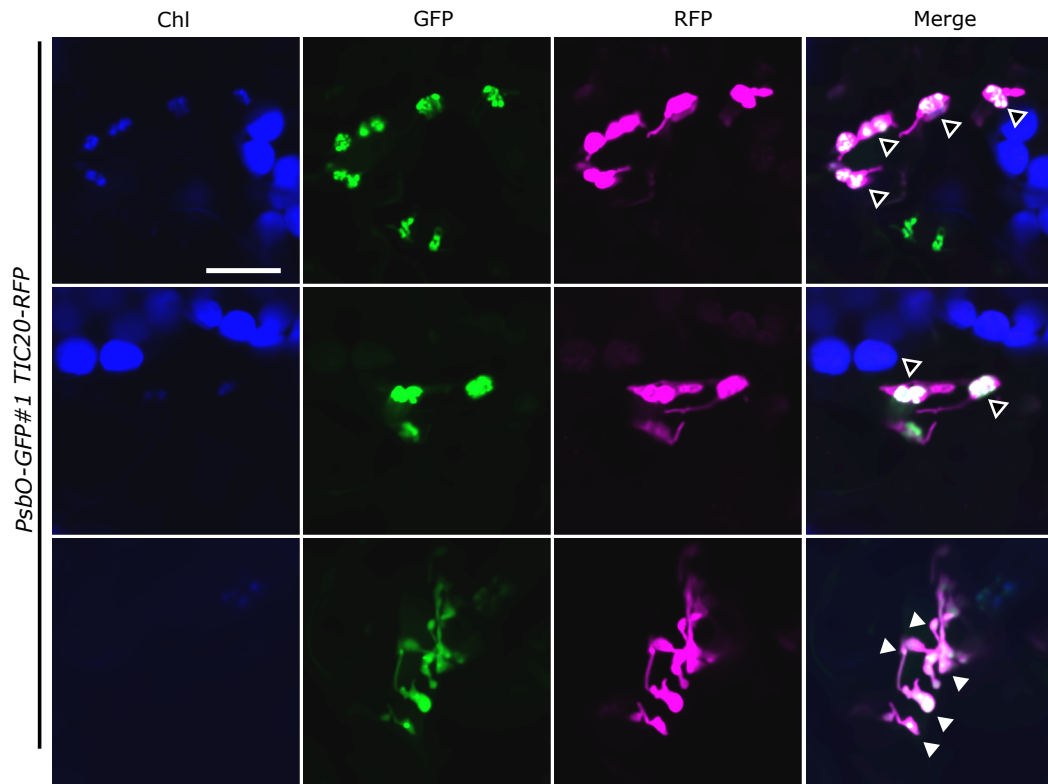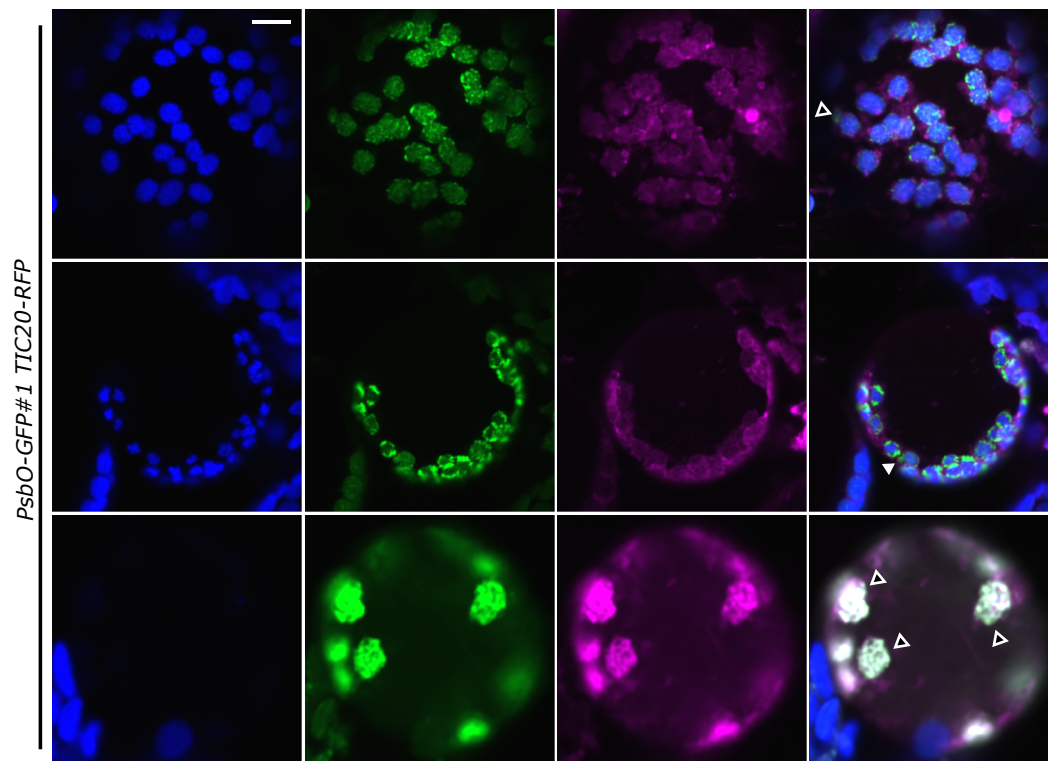

Supplement: Supplementary file 9 — Figure S9. [file PCE-48-6373-s014.pdf]

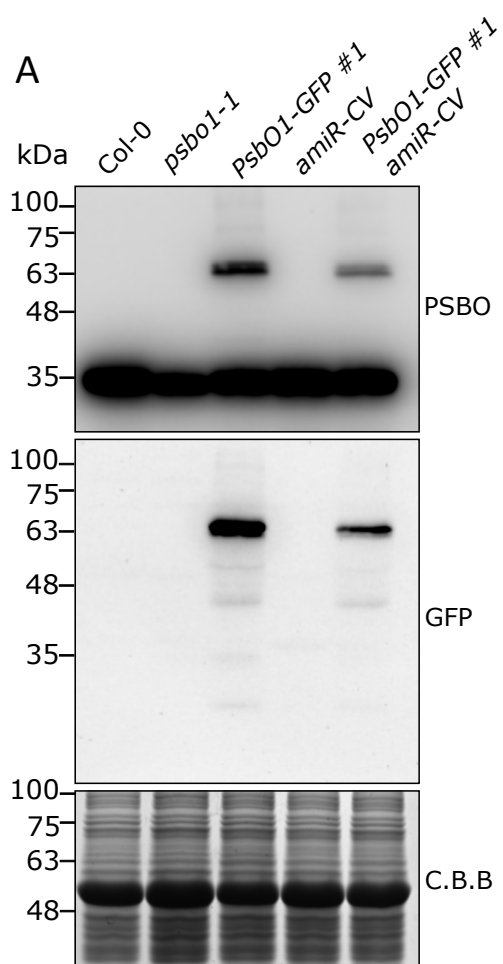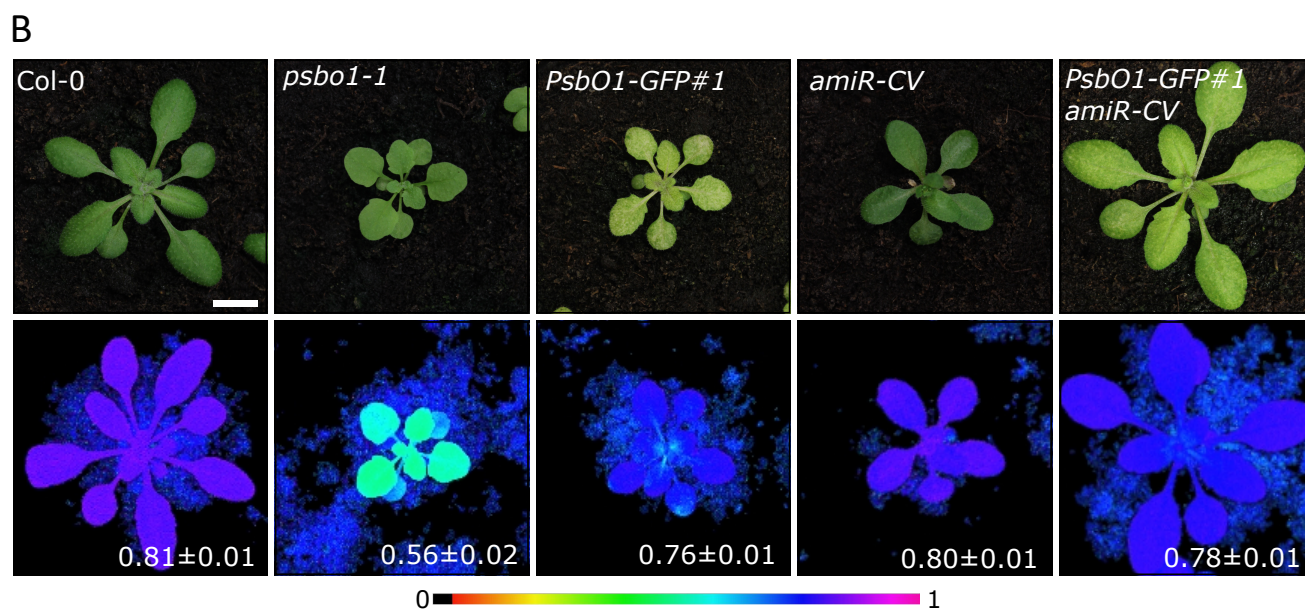

Supplement: Supplementary file 10 — Figure S10. [file PCE-48-6373-s010.pdf]
