# Supplementary material for: A Holistic Investigation of Arabidopsis Proteomes Altered in Chloroplast Biogenesis and Retrograde Signalling Identifies PsbO as a Key Regulator of Chloroplast Quality Control
Source: Plant Cell Environ. 2025 May 14;48(8):6373–96. doi: 10.1111/pce.15611 (PMC12223713; doi:10.1111/pce.15611)
Supplement: Supplementary file 18 — supmat. [file PCE-48-6373-s008.docx]

**Figure S1.** Proteomes from *Arabidopsis thaliana* Col-0 and *gun1* seedlings grown on MS medium in absence or presence (-/+) of Lin. Spearman’s correlation between pairs of **A**) Col-0, **B**) Col-0+Lin, **C**) *gun1* and **D**) *gun1*+Lin biological replicates. **E**) 2D-Map (pI vs MW) of the total distinct identified proteins (n = 9024) and distribution of the average Spectral Count (Av.SpC) per protein (blue dots, Av.SpC < 1; yellow dots, 1 ≤ Av.SpC < 10; red dots, Av.SpC ≥ 10. **F**) Spearman’s correlation among the average proteome profiles of Col-0 (n = 9), Col-0+Lin (n = 9), *gun1* (n = 9) and *gun1*+Lin (n = 9). **G**) Principal Component Analysis (PCA) of selected DAPs with p ≤ 0.001. **H**) Histogram showing the number of DAPs found (LDA, p ≤ 0.01) per comparison (*gun1* *vs* *gun1*+Lin; Col-0 *vs* Col-0+Lin; Col-0+Lin *vs* *gun1*+Lin; Col-0 *vs* *gun1*).

**Figure S2**. Biological Processes (BP) and Molecular Functions (MF) GO terms differentially enriched in *A. thaliana* Col-0 and *gun1* mutant seedlings grown with or without lincomycin (+/-Lin). **A**) BPs were retrieved from the TAIR and PANTHER databases, while those differentially enriched were extracted by using LDA (*n* = 9 per condition, *p* < 0.01, *F* ratio > 4.8). **B**) MFs were retrieved from TAIR and PANTHER databases, while those differentially enriched were extracted by using LDA (*n* = 9 per condition, *p* < 0.01).

**Figure S3**. Graphical representation of Protein-Protein Interaction (PPI) network modules detected in the different proteome profiles (**A** Col-0; **B** *gun1*; **C** Col-0 +Lin; **D** *gun1* +Lin). The network was reconstructed from STRING db starting from high-confidence differentially abundant proteins (DAPs; *p* ≤ 0.001). The network is comprised of 326 nodes and 2479 edges. Protein accumulation is represented by normalized spectral count (nSpC) values (range 0-100). Blue and red nodes indicate down- and up-regulated proteins, respectively.

**Figure S4**. Selection of DAPs up-regulated in the presence of Lin. These proteins are functionally involved in fatty-acid metabolism, oxidative stress response, protein folding, vesicle transport (ER-Golgi), organelle transport, vacuolar processes, and proteolysis. For each protein, the normalized spectral count value (nSpC) in the range 0-100 is shown (LDA, *p* ≤ 0.01).

**Figure S5**. PPI and co-expression network models of Col-0 and *gun1* seedlings grown in the absence and presence of Lin (-/+Lin). **A**) Degree distribution of Col-0, *gun1*, Col-0+Lin and *gun1*+Lin co-expression network models. The violin plots report the average betweenness in the corresponding random network models. **B**) Degree distribution of Col-0, *gun1*, Col-0+Lin and *gun1*+Lin co-expression network models. Here, the violin plots report the average Degree in the corresponding random network models.

**Figure S6**. Mitochondrial morphology in the absence and presence of Lin (-/+ Lin). Confocal microscopy showing the distributions of the YFP protein (mTP-YFP) in mitochondria of Col-0 and *gun1-102* genetic backgrounds, grown in presence or absence of Lin (-/+Lin).

**Figure S7**. PsbO accumulation in the presence of Lin. **A**) Immunoblot analyses of Col-0, *gun1-102* and *psbq1-1 psbq2-1 psbr-1* (-/+Lin) total protein extracts probed with PsbR- and PsbQ-specific antibodies. Coomassie Brilliant Blue (C.B.B.) staining of SDS-PAGE is shown as loading control. **B**) Immunoblot analyses of total protein extracts (tot) and enriched plastid fractions (p) from Col-0 and *gun1-102* samples grown in presence of Lin were performed. Anti-HSP90 antibodies were used as a marker for extra-plastid fraction, while anti-CPN60 antibodies specifically mark the plastid fraction. Anti-PsbO antibodies demonstrate the exclusive presence of PsbO precursors in the exta-plastid fraction. **C**) Real-time quantitative PCR analyses of the expression of subunits of the Oxygen Evolving Complex subunits *PsbO*, *PsbQ*, *PsbP* and *PsbR* in Col-0, *gun1-102*, *psbo1-1* and *gun1-102 psbo1-1* (-/+Lin) genotypes **D**) Transmission electron micrographs of mesophyll cells from 6 DAS seedlings of the indicated genotypes grown on MS medium with 55 µM Lin. Legend: thy, thylakoids; pg, plastoglobuli; ve, vesicle; bv, budding vesicles; dp. degradation products; cv, collapsing vacuole. The scale bar represents 1 µm.

**Figure S8**. PsbO involvement in chloroplast quality control and degradation. **A**) Immunoblot analyses of total protein extracts from Col-0, *psbo1-1* and the two independent *oePSBO1-GFP* lines were carried out at 12 DAS, and blots were probed with PsbO- and GFP-specific antibodies. Coomassie Brilliant Blue (C.B.B.) staining of SDS-PAGE is shown as loading control. Asterisk (*) and arrowhead (<) indicate the GFP constructs and the endogenous PsbO, respectively. Numbers indicate the PsbO signal intensities relative to Col-0. **B)** Images of visible phenotypes and Chl fluorescence of the indicated genotypes obtained from 18 DAS plants. The fluorometric data depict the *Fv/Fm* parameter in false colours, together with the average ± SD values (*n* ≥4). Scale bar: 1 cm. **C**) Images of visible phenotypes of 6DAS seedlings grown on MS medium in absence or presence of 4 µM DEX. **D**) Immunoblot analyses of total protein extracts from leaf-discs harvested from two independent *indPsbO1-GFP* lines and incubated for the indicated amount of time (HAI, hours after induction) in presence of DEX. Blots were probed with PsbO- and GFP-specific antibodies. Coomassie Brilliant Blue (C.B.B.) staining of PVDF filter is shown as loading control. Asterisk (*) and arrowhead (<) indicate the GFP constructs and the endogenous PsbO,

**Figure S9.** Fluorescence signals from chlorophylls (Chl, blue), GFP (green) and RFP (magenta) detected in mesophyll tissue (upper panel) and protoplasts (lower panel) obtained from the *PsbO1-GFP#1 TIC20-RFP* line. Solid arrowheads indicate vesicles with GFP and RFP fluorescence, while empty arrowheads highlight chloroplasts showing GFP, RFP and Chl fluorescence. Scale bar: 10 µm.

**Figure S10**. Functional interaction of PsbO with CV. **A**) Immunoblot analyses of total protein extracts from Col-0, *psbo1-1*, *PsbO1-GFP#1*, *amiR-CV* and the *PsbO1-GFP#1* *amiR-CV* double mutant. Plants were grown on soil, and probed with PsbO- and GFP-specific antibodies at 12 DAS. Coomassie Brilliant Blue (C.B.B.) staining of SDS-PAGE is shown as loading control. **B**) Visible phenotypes of the indicated genotypes and fluorometric images representing the *Fv/Fm* parameter in false colours and the average ± SD values (*n* ≥ 4). Scale bar: 1 cm.

**Table S1.** Proteins identified in *A. thaliana* Col-0 and the *gun1* mutant, grown with and without lincomycin (+/-Lin). For each protein, the normalized Spectral Count (SpC) per biological replicate (rep) is shown, as well as the average SpC (Av.SpC) per condition. IF: Identification Frequency. Proteins found in all 36 replicates are highlighted in grey.

**Table S2**. Differentially enriched GO Biological Process terms (BPs) identified by comparing the enrichment profiles (by both fold-enrichment and numbers of proteins) obtained from Col-0, Col-0+Lin, *gun1* and *gun1*+Lin (n = 9 per condition). BPs were retrieved from TAIR and PANTHER databases, while those differentially enriched were extracted by Linear Discriminant Analysis (LDA). *p* ≤0.01, *F* ratio = LN(SpC1/SpC2).

**Table S3**. A) Differentially Abundant Proteins (DAPs) detected in *A. thaliana* Col-0 and *gun1* mutant seedlings grown with and without lincomycin (+/-Lin). Proteins were selected by LDA (*p* < 0.05). The most significant DAPs (*p* < 0.01) are highlighted in bold. Fold change was calculated as FC = LN(SpC1/SpC2)., Positive values (red) indicate proteins up-regulated under the first condition (red), while negative values (blue) indicate proteins up-regulated under the second condition (blue). B) Subset of the proteins listed in A in which the absolute protein abundance was estimated by normalization using the average amino-acid molecular weight.

**Table S4**. PPI network hubs that characterize the proteomes obtained from *A. thaliana* Col-0 and *gun1* seedlings grown on MS medium with and without lincomycin (+/-Lin). Proteins were defined as hubs if Betweenness, Centroid and Bridging values were higher than the average values calculated for whole-network models (in bold, black); while centrality values that do not exceed the imposed thresholds are indicated in red. Centralities values used to identify hubs characterizing specific phenotypes are highlighted in yellow. For each hub, the average Spectral Count and the fold change (if DAP, too) are shown. A) Hubs detected under all conditions. B) Hubs detected in Col-0. C) Hubs detected in *gun1*. D) Hubs in Col-0+Lin. E) Hubs in *gun1*+Lin. F) Hubs that are characteristic for growth on Lin. G) Hubs that characterize *gun1*.

**Table S5**. Co-expression network hubs that characterize the proteomes obtained from *A. thaliana* Col-0 and *gun1* seedlings grown on MS medium with and without lincomycin (+/-Lin). Proteins were defined as hubs if the Degree value was higher than the average values calculated for whole network models (in bold, black) and less than the average in the other conditions. For each protein, GO Cellular Component is shown. Degree values used to identify differentially co-expressed hubs characterizing specific phenotypes are highlighted in yellow. A) Hubs in Col-0, B) Hubs in *gun1*, C) Hubs in Col-0+Lin, D) Hubs in *gun1*+Lin. E) Hubs in Col-0 and Col-0+Lin. F) Hubs in *gun1* and *gun1*+Lin, G) Hubs in Col-0 and *gun1*, H) Hubs in Col-0+Lin and *gun1*+Lin.

**Table S6**. Tryptic peptides identified by LC-MS/MS analysis in PsbO bands of higher molecular weight (+ and * bands, respectively) identified by immunoblot analyses on total protein extract from seedlings grown in presence of 550 µM Lin probed with PsbO antibody. Peptides that were mapped to chloroplast transit-peptide sequences (residues 1 to 58) are shown in blue, and peptides mapping to thylakoid transit-peptide sequences (residues 59 to 85) are indicated in red.

**Table S7**. Oligonucleotide sequences employed for RT-qPCR and cloning.
